# Ultralarge Stokes Shift Phosphorescence Artificial Harvesting Supramolecular System with Near‐Infrared Emission

**DOI:** 10.1002/advs.202201523

**Published:** 2022-06-02

**Authors:** Man Huo, Xian‐Yin Dai, Yu Liu

**Affiliations:** ^1^ College of Chemistry State Key Laboratory of Elemento‐Organic Chemistry Nankai University Tianjin 300071 P. R. China

**Keywords:** near‐infrared cell labeling, near‐infrared delayed emission, phosphorescence, phosphorescence artificial harvesting, two‐step sequential energy transfer

## Abstract

A two‐step sequential phosphorescence harvesting system with ultralarge Stokes shift and near‐infrared (NIR) emission at 825 nm is successfully constructed by racemic 1,2‐diaminocyclohexan‐derived 6‐bromoisoquinoline (BQ), cucurbit[8]uril (CB[8]), and amphipathic sulfonatocalix[4]arene (SC4AD) via cascaded assembly strategy in aqueous solution. In virtue of the confinement effect of CB[8] with rigid cavity, BQ can generate an emerging phosphorescent emission at 555 nm. Subsequently, the binary BQ⊂CB[8] further assemblies with SC4AD to form close‐packed spherical aggregate, which contributes to the dramatic enhancement of phosphorescence emission intensity ≈30 times with prolonged lifetime from 21.3 µs to 0.364 ms. Notably, the BQ⊂CB[8]@SC4AD assembly can serve as an energy donor to conduct stepwise phosphorescence harvesting process through successive introduction of primary acceptors, cyanine 5 (Cy5) or nile blue (NiB), and secondary acceptor, heptamethine cyanine (IR780). The final aggregate with remarkable ultralarge Stokes shift (≈525 nm) and long‐lived NIR photoluminescence (PL) emission at 825 nm is further employed as imaging agent for NIR cell labeling.

## Graphical abstract

A stepwise phosphorescence artificial harvesting supramolecular system is constructed using 1,2‐diaminocyclohexan‐derived 6‐bromoisoquinoline, cucurbit[8]uril, and amphipathic sulfonatocalix[4]arene in aqueous solution through successively introducing primary acceptors (Cy5  or NiB) and final acceptor (IR780), presenting ultralarge Stokes shift (≈525 nm) and long‐lived NIR photoluminescence emission at 825 nm, thus being successfully applied for NIR cell labeling.

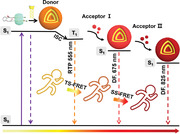

## 1 Introduction

Organic NIR materials have garnered considerable attention due to their superior penetrability, higher spatial distinguishability, and lower light scattering in bioimaging.^[^ ^1^ ^]^ To date, a variety of strategies have been developed to fabricate NIR emissive materials, such as the extension the *π*‐conjugate for unsaturated structure,^[^ ^2^ ^]^ combination of the electron‐donor and acceptor parts,^[^ ^3^ ^]^ polymerization,^[^ ^4^ ^]^ and cocrystallization.^[^ ^5^ ^]^ In particular, supramolecular assembly strategy based on host–guest induced emission and assembling‐induced emission, has evolved as an applicable and more powerful approach for construction of NIR emission nanosystem.^[^ ^6^ ^]^ Cavity‐bearing macrocycles (e.g., cucurbit[*n*]urils,^[^ ^7^ ^]^ pillar[*n*]arenes^[^ ^8^ ^]^) can significantly regulate the aggregation behaviors of organic guests to realize the NIR emissive performance. Moreover, the well‐defined supramolecular assemblies can function as the viable scaffolds to create the light‐harvesting systems through introducing appropriated acceptors, thereby achieving large Stokes redshifted emission.^[^ ^9^ ^]^

In comparison with fluorescent materials, NIR‐phosphorescent materials are of particular interests benefiting from their long lifetime which can mitigate the interference of background fluorescence or autofluorescence.^[^ ^10^ ^]^ However, such materials are hard to acquire because of the limitation by the energy gap law.^[^ ^11^ ^]^ Recently, phosphorescence resonance energy transfer (PRET) has emerged as an alternative which can endow suitable fluorescent organic dyes with long lifetime via a delayed sensitization process,^[^ ^12^ ^]^ showing greatly potential applications in time‐resolved bioimaging,^[^ ^13^ ^]^ multicolor afterglow materials,^[^ ^14^ ^]^ information encryption, and anticounterfeiting.^[^ ^15^ ^]^ For example, George and coworkers have reported an organic–inorganic scaffolding strategy which can be used as a light‐harvesting platform to achieve delayed fluorescence via PRET process.^[^ ^16^ ^]^ In contrast to single one‐step energy transfer process, cascaded energy transfer exhibits large Stokes shift from initial donors to bathochromic‐shift acceptors, tackling the invalid spectral overlap issue between them by the assistance of the intermediate acceptors.^[^ ^17^ ^]^ Importantly, the triplet‐involved stepwise energy transfer (TSS‐FRET) has been proposed to formulate the color‐tunable room‐temperature phosphorescence (RTP) emission system.^[^ ^18^ ^]^ More recently, Chi et al. reported a remarkable persistent NIR luminescence (810 nm) system in the solid‐state via stepwise energy transfer based on successively doping two organic dyes (NiB and cyanine 7) into the triphenylene‐contained polymer.^[^ ^19^ ^]^ Thus so far, although great progress has been made in the fabrication of such systems in solid state, the stepwise phosphorescence harvesting system possessing ultralarge Stokes shift with long wavelength (over 800 nm) has not been reported in aqueous solution, to the best of our knowledge.

**Scheme 1:**
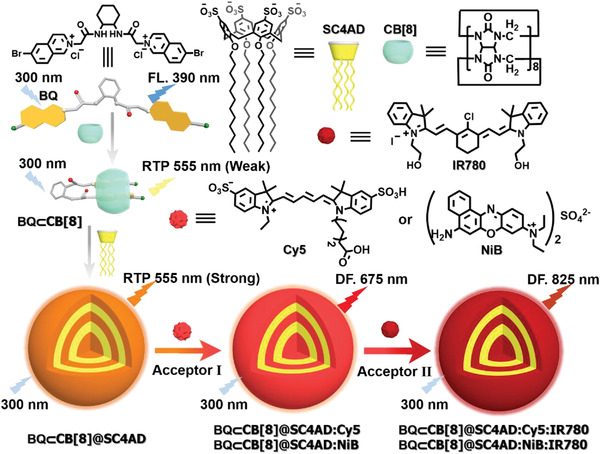
Schematic illustration of the construction of two‐step sequential phosphorescence harvesting system with ultralarge Stokes shift and long‐lived NIR emission in aqueous solution.

Herein, we reported a high‐efficiency two‐step sequential phosphorescence harvesting system featuring ultralarge Stokes shift (≈525 nm) and long‐lived NIR emission at 825 nm in aqueous solution, which was constructed from BQ, CB[8] and amphiphilic SC4AD via cascaded assembly strategy (**Scheme** **1**). The initial guest (BQ) generated a weak phosphorescent emission at 555 nm by host–guest interaction with CB[8], which further assembled with SC4AD to form homogeneously spherical nanoparticles with the dramatical enhancement of both phosphorescence lifetime to 0.364 ms and emission intensity by 30‐fold. The hydrophobic interior of such compact assembly BQ⊂CB[8]@SC4AD not only protected the phosphor from the influence of quenchers to a certain extent, but also provided a microenvironment for loading the fluorescent dyes to perform the effective energy transfer. Benefiting from the good overlap between the phosphorescence emission region of BQ⊂CB[8]@SC4AD assembly and the absorption band of Cy5 or NiB, the first‐step energy transfer process took place from the triplet‐state of donor to the singlet‐states of acceptors (Cy5 or NiB). Followed by the doping of secondary dye (IR780) into above systems, second‐step energy transfer process occurred from Cy5 or NiB to IR780 (BQ⊂CB[8]@SC4AD:Cy5:IR780 = 90:3:1, *Ф*_ET1_ = 46.9%, *Ф*_ET2_ = 48.6%; BQ⊂CB[8]@SC4AD:NiB:IR780 = 900:20:9, *Ф*_ET1_ = 52.2%, *Ф*_ET2_ = 45.5%), which finally displayed long‐lived NIR luminescence at 825 nm from final acceptor and were successfully applied in cell labeling. The stepwise phosphorescence harvesting approach offers a novel method for the creation of the long‐lived NIR emissive system with ultralarge Stokes shift in aqueous solution.

## 2 Results and Discussion

**Figure 1:**
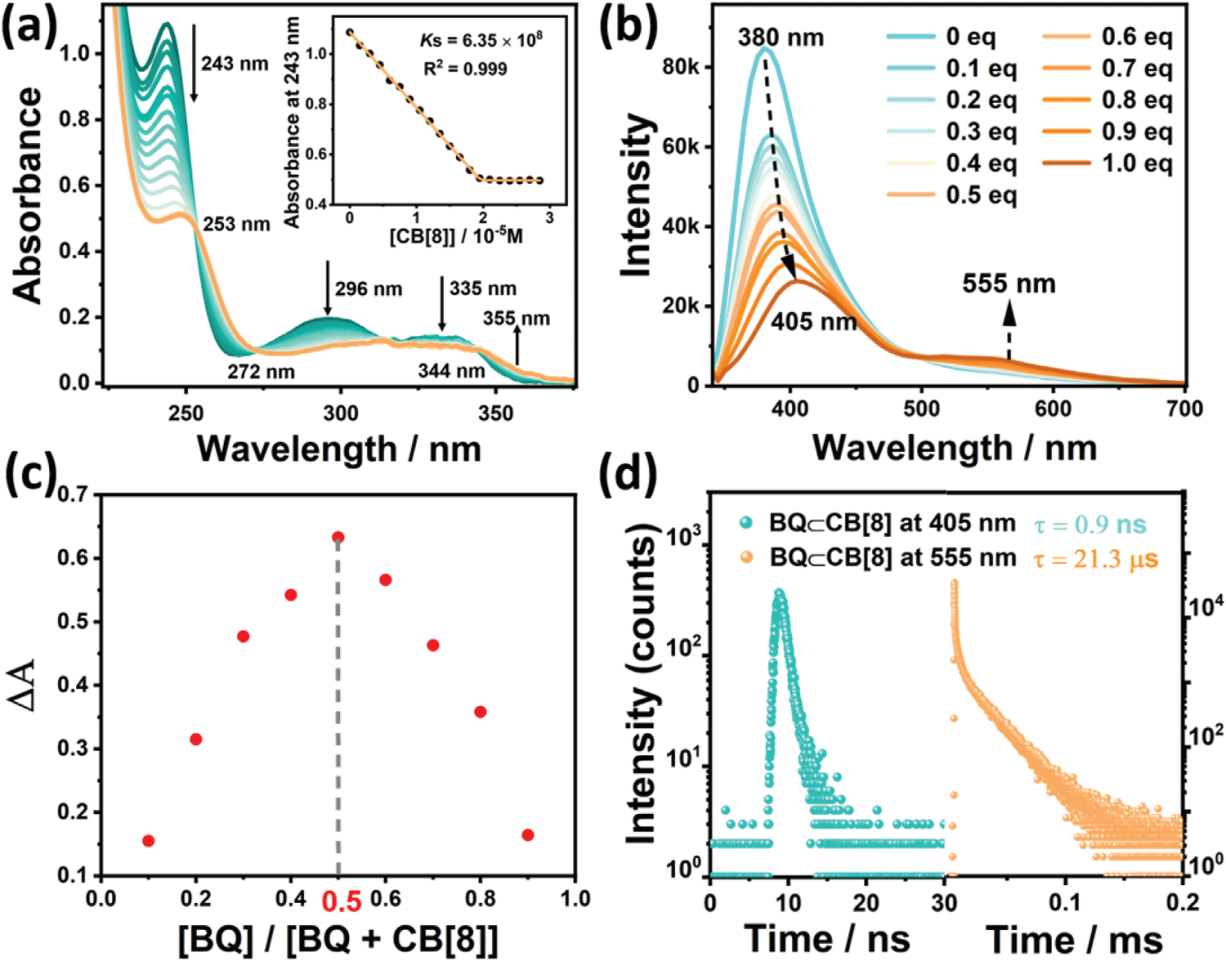
a) UV–vis absorption spectra and absorbance intensity changes of BQ at 243 nm (inset) upon addition of CB[8] in H_2_O at 298 K ([BQ] = 1.5 × 10^−5^ m and [CB[8]] = 0−3.0 × 10^−5^ m). b) PL emission spectral changes of BQ upon addition of 0, 0.1, 0.2, 0.3, 0.4, 0.5, 0.6, 0.7, 0.8, 0.9, and 1.0 equivalent CB[8] in H_2_O at 298 K ([BQ] = 3.0 × 10^−5^ m, *λ*_ex_ = 300 nm). c) Job's‐plot showing the 1:1 stoichiometry of the complex between BQ and CB[8] by UV titration. d) Time‐resolved PL decay spectra of BQ⊂CB[8] at 405 and at 555 nm in H_2_O at 298 K.

It has been proved that 6‐bromoisoquinoline derivatives can produce the desired CB[7]‐induced RTP in aqueous solution.^[^ ^20^ ^]^ Herein, the phosphorescent chromophore, BQ was prepared via the salt formation reaction between racemic nonfluorescent core 1,2‐diaminocyclohexane and 6‐bromoisoquinoline, and the compound characterizations are shown in the Figures S1–S3, Supporting Information. First, UV–vis spectroscopy was conducted to investigate the host–guest binding behavior between BQ and CB[8]. As illustrated in **Figure** **1**a, free BQ displayed three absorption peaks at 243, 296, and 335 nm, respectively. Upon stepwise addition of CB[8], the original absorption peaks underwent bathochromic shift and the intensity decreased, followed by the appearance of three isosbestic points at 253, 272, and 344 nm. These observations suggested that *π*–*π* stacking of two 6‐bromoisoquinoline in the cavity of CB[8] could give rise to the host‐stabilized intermolecular charge transfer interaction.^[^ ^21^ ^]^ Moreover, the Job's plot validated that the binding stoichiometric ratio was 1:1 between BQ and CB[8] (Figure 1c). The association constant (*K*_a_) in BQ⊂CB[8] was determined as 6.35 × 10^8^ in aqueous solution by nonlinear least‐squares method (Figure 1a, inset). Additionally, the resultant 1:1 binary species BQ⊂CB[8] was evaluated by ^1^H NMR titration experiments. With the addition of CB[8], the aromatic proton signals (H_c–h_) underwent the upfield shift due to the shielding effect of CB[8] cavity, while the protons of the diaminocyclohexane moiety (H_a,b_) of BQ shifted to downfield (Figure S6, Supporting Information). More importantly, the protons on the two 6‐bromoisoquinoline of BQ displayed different signals, which may be probably caused by discrepant chemical environment. Thus, we speculated that two parts of 6‐bromoisoquinoline entered the CB[8] cavity in a dislocation stacking mode. And it was found that the proton chemical shift changes of BQ came to equilibrium state with 1.0 equivalent CB[8]. In addition, 2D rotating‐frame overhauser effect spectroscopy exhibited obvious correlation peaks between two parts of 6‐bromoisoquinoline, further corroborating the dislocation stacking mode for BQ⊂CB[8] (Figure S7, Supporting Information). To gain more insights into the assembling mode, 2D diffusion‐ordered spectroscopy was conducted, which showed that the diffusion coefficients (D) were 3.328 × 10^−10^ to 2.424 × 10^−10^ for BQ and BQ⊂CB[8], implying that there was no significant dimensional change before and after being encapsulated by CB[8] for BQ (Figure S8, Supporting Information). Meanwhile, there was no fibrous nano‐aggregates observed in the transmission electron microscopic (TEM) image, excluding the formation of supramolecular polymers for BQ⊂CB[8] complex (Figure S9, Supporting Information). Importantly, mass spectrometry was also carried out to study the host–guest inclusion complex BQ⊂CB[8], and the intense m/z peak at 971.064 could be assigned to [BQ+CB[8]‐2Cl]^2+^ (Figure S10, Supporting Information).^[^ ^22^ ^]^ Taken together, these results substantiated the formation of 1:1 foldamer‐like dislocation stacking complexation between BQ and CB[8] in aqueous solution driven by host‐enhanced intermolecular charge transfer interaction.

**Figure 2:**
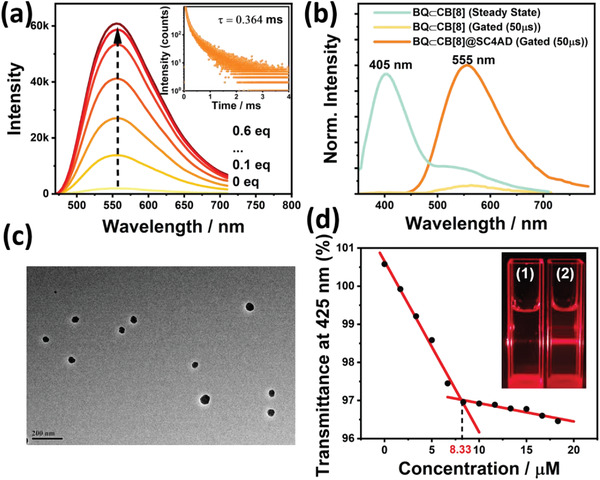
a) Phosphorescence emission spectra (delayed 50 µs) of BQ⊂CB[8] at 555 nm upon addition of 0, 0.1, 0.2, 0.3, 0.4, 0.5, and 0.6 equivalent SC4AD in H_2_O at 298 K ([BQ] = 3.0 × 10^−5^ m, *λ*_ex_ = 300 nm). Inset: Time‐resolved PL decay spectra of BQ⊂CB[8]@SC4AD at 555 nm in H_2_O at 298 K ([BQ] = 3.0 × 10^−5^ m, [CB[8]] = 3.0 × 10^−5^ m, [SC4AD] = 1.5 × 10^−5^ m). b) The prompt PL spectrum of BQ⊂CB[8], phosphorescence emission spectra (delayed 50 µs) of BQ⊂CB[8] and BQ⊂CB[8]@SC4AD in H_2_O at 298 K ([BQ] = 3.0 × 10^−5^ m, [CB[8]] = 3.0 × 10^−5^ m, [SC4AD] = 1.5 × 10^−5^ m). c) Transmission electron microscopy image of BQ⊂CB[8]@SC4AD assembly. d) Dependence of the optical transmittance at 425 nm upon addition of SC4AD. Inset: Tyndall effects of BQ⊂CB[8] (1) and BQ⊂CB[8]@SC4AD (2).

Subsequently, the optical proprieties of BQ⊂CB[8] in aqueous solution was studied, especially its CB[8] macrocycle‐confined phosphorescence behavior. As discerned from Figure 1b, the emission peak of free BQ appeared at 390 nm, and a new emission peak would arise at 555 nm accompanied by the red shift of the early peak from 390 to 405 nm with addition of CB[8]. With a delay time of 50 µs, the emission peak at 405 nm vanished and only the emission peak at 555 nm maintained in the phosphorescence spectra, as an indication of the existence of host‐induced long‐lived species (**Figure** **2**b). At the same time, the lifetime at 405 and 555 nm were measured, respectively, by time‐resolved PL decay curves (Figure 1d), which revealed that the former was on the order of nanoseconds (0.9 ns) which was regarded as short‐lived fluorescent emission, while the latter was on the order of microseconds (21.3 µs) on behalf of a kind of long‐lived species. Moreover, the emission intensity at 555 nm would increase after blowing with nitrogen in aqueous solution (Figure S11, Supporting Information), and the lifetime extended to 0.204 ms (Figure S12, Supporting Information) as a result of the triplet‐state excitons quenching being suppressed. Furthermore, the emission intensity at 555 nm was substantially increased at 77 K (Figure S13, Supporting Information). The phosphorescence quantum yield of BQ⊂CB[8] was determined to be 0.75% (Figure S14, Supporting Information). Thus, it could be concluded that the emission at 555 nm for BQ⊂CB[8] was considered to be CB[8]‐induced phosphorescence in aqueous solution, and the red‐shifted phosphorescence emission was due to the host‐stabilized ICT interaction attributed to 1:1 foldamer‐like dislocation stacking BQ⊂CB[8] complexation.^[^ ^23^ ^]^

Benefiting from its dodecyl‐modified upper portals and sulfonate‐laced lower portals, SC4AD with pre‐organized amphiphilic structure can serve as important building block for constructing the emissive enhancement nanosystems.^[^ ^24^ ^]^ Herein, SC4AD was utilized to co‐assemble with BQ⊂CB[8] for gaining the ternary BQ⊂CB[8]@SC4AD assembly in aqueous solution. As depicted in Figure 2a, the preeminent enhancement of phosphorescence emission at 555 nm was observed after gradual addition of SC4AD into BQ⊂CB[8] solution, which reached equilibrium state with 0.5 equiv. SC4AD, implying that co‐assembly with SC4AD engender more effective phosphorescence emission for BQ⊂CB[8] in aqueous media. In contrast with BQ⊂CB[8], BQ⊂CB[8]@SC4AD assembly showed a significant increase in phosphorescent quantum yield of 5.46% (Figure S15, Supporting Information). In addition, the time‐resolved PL decay curve analysis demonstrated that the phosphorescence lifetime for the resultant ternary BQ⊂CB[8]@SC4AD assembly at 555 nm extremely extended to 0.364 ms (Figure 2a, inset). These phenomena suggested that profiting from its inherent amphiphilicity, co‐assembly with SC4AD could facilitate the singlet‐triplet ISC and provided a phosphor vibration‐limited hydrophobic microenvironment to immensely suppress nonradiative transition from triplet to singlet by quenchers. As judged from Figure 2b, it seemed pretty obvious that with a delay time of 50 µs, the phosphorescence spectrum of BQ@SC4AD was silent (Figure S16, Supporting Information), while phosphorescence spectrum of BQ⊂CB[8] showed a faint emission peak at 555 nm. As for BQ⊂CB[8]@SC4AD assembly, there was a dramatical enhancement in intensity at the same experimental conditions. These results jointly illustrated that introduction of SC4AD could definitely promote phosphorescence emissive performance on the foundation of CB[8]‐induced phosphorescence emission. In another contrast experiment, the phosphorescence emission at 555 nm presented imperceptible increase after the stepwise addition of sodium dodecyl benzene sulfonate (Figure S17, Supporting Information), which confirmed that the amphiphilic multicharge pre‐organized structure for SC4AD acted a crucial role in highly efficient co‐assembly with BQ⊂CB[8] to elevate the phosphorescent emission in aqueous solution.

To shed more light on the self‐assembly behavior between BQ⊂CB[8] and SC4AD, transmittance experiments were performed to monitor the changes of optical transmittance under different concentration of SC4AD. As shown in Figure 2d, the critical aggregation concentration was determined as 8.33 µm judging from the transmittance changes at 425 nm (Figure S18, Supporting Information). Furthermore, more detailed morphologic structures of BQ⊂CB[8]@SC4AD assembly were measured by making use of TEM and dynamic light scattering (DLS). TEM image displayed that BQ⊂CB[8]@SC4AD self‐assembled to some homogeneous near‐spherical shape nanoparticles with diameter around 70 nm (Figure 2c). In addition, BQ⊂CB[8]@SC4AD demonstrated more recognizable Tyndall effect in comparison with BQ⊂CB[8] complex, further validating aforementioned conclusion of formation of large‐sized assemblies (Figure 2d, inset). Meanwhile, DLS experiments showed that the majority of nanoparticles were distributed in 50–≈70 nm in diameter and there was a little of nanoparticles with diameter of ≈110 nm, consistent with the TEM results (Figure S19, Supporting Information). Given all of that, SC4AD‐participated ternary supramolecular assembly was favorably constructed and attributed to synergistic force including hydrophobic interaction and electrostatic interaction.

**Figure 3:**
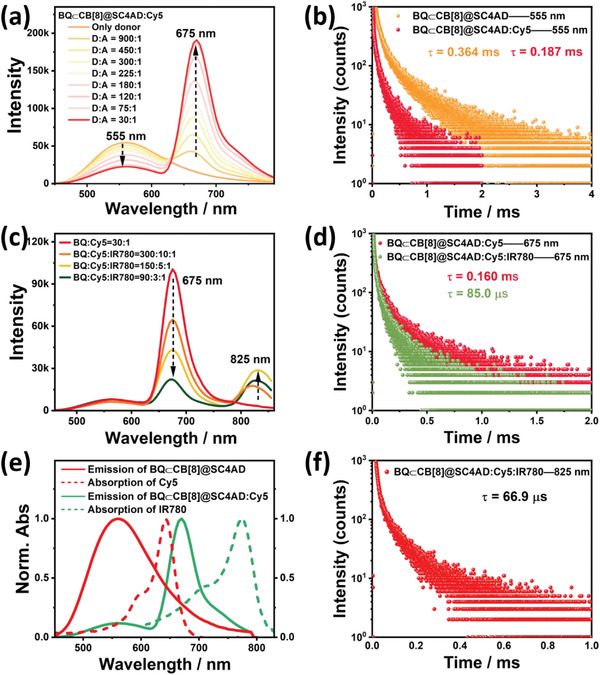
a) Phosphorescence emission spectra (delayed 50 µs) of BQ⊂CB[8]@SC4AD:Cy5 at different donor/acceptor ratios in aqueous solution at 298 K ([BQ⊂CB[8]] = 3.0 × 10^−5^ m, [SC4AD] = 1.5 × 10^−5^ m, *λ*_ex_ = 300 nm). b) Time‐resolved PL decay curves of BQ⊂CB[8]@SC4AD, BQ⊂CB[8]@SC4AD:Cy5 at 555 nm in aqueous solution at 298 K ([BQ⊂CB[8]] = 3.0 × 10^−5^ m, [SC4AD] = 1.5 × 10^−5^ m, [Cy5] = 1.0 × 10^−6^ m). c) Phosphorescence emission spectra (delayed 50 µs) of BQ⊂CB[8]@SC4AD:Cy5:IR780 at different donor/acceptor ratios in aqueous solution at 298 K ([BQ⊂CB[8]] = 3.0 × 10^−5^ m, [SC4AD] = 1.5 × 10^−5^ m, [Cy5] = 1.0 × 10^−6^ m, *λ*_ex_ = 300 nm). d) Time‐resolved PL decay curves of BQ⊂CB[8]@SC4AD:Cy5, BQ⊂CB[8]@SC4AD:Cy5:IR780 at 675 nm in aqueous solution at 298 K ([BQ⊂CB[8]] = 3.0 × 10^−5^ m, [SC4AD] = 1.5 × 10^−5^ m, [Cy5] = 1.0 × 10^−6^ m, [IR780] = 3.33 × 10^−7^ m). e) Normalized emission spectrum of BQ⊂CB[8]@SC4AD (red solid line) and BQ⊂CB[8]@SC4AD:Cy5 (green solid line) assembly and absorption spectra of Cy5 (red short dash), IR780 (green short dash). f) Time‐resolved PL decay curves of BQ⊂CB[8]@SC4AD:Cy5:IR780 at 825 nm in aqueous solution at 298 K ([BQ⊂CB[8]] = 3.0 × 10^−5^ m, [SC4AD] = 1.5 × 10^−5^ m, [Cy5] = 1.0 × 10^−6^ m, [IR780] = 3.33 × 10^−7^ m).

Cascaded FRET is a novel strategy which implicitly needs to go through two‐step energy transfer process so as to resolve the invalid spectral overlap between donors and bathochromic‐shift organic dyes by the aid of the intermediate acceptors, thus realizing the organic NIR luminescent system.^[^ ^25^ ^]^ RTP‐based cascaded FRET (TSS‐FRET) has mainly undergone two consecutive phases: TS‐FRET and SS‐FRET, for the sake of broadening the spectral range of long‐lived species.^[^ ^18^ ^]^ TS‐FRET originated in fluorescence‐based FRET process between singlet‐states of different chromophores, takes place from the triplet‐state of phosphors to the singlet‐state of organic dyes, which arouses the burgeoning of multicolor long‐lived luminescent material based on this strategy.^[^ ^26^ ^]^ It was a crucial matter for TTS‐FRET process to select the appropriate receptor. In view of our system, the BQ⊂CB[8]@SC4AD aggregates, on one hand, behaved greatly host‐induced and assembly‐enhanced phosphorescent characteristics to make sure to be as a high‐performing light‐harvesting scaffold. On the other hand, it possessed an amphiphilic multicharge pre‐organized stacking structure, which provided an internal hydrophobic environment for loading the organic dyes, thus shortening the distance between donors and acceptors and improving the luminous efficiency of dyes in aqueous phases. Cy5, a universal commercial organic dye (*λ*_em_ = 675 nm) with high quantum yield, was selected as acceptors owing to its absorption band having a valid overlap with the emission of BQ⊂CB[8]@SC4AD aggregates (**Figure** **3**e). The obtained Cy5‐loaded BQ⊂CB[8]@SC4AD assembly displayed a distinctly increased average diameter (Figure S20, Supporting Information) compared with BQ⊂CB[8]@SC4AD. As illustrated in Figure 3a, the Cy5‐added systems (BQ⊂CB[8]@SC4AD:Cy5) presented a markedly rising trend of emission peak at 675 nm in the delayed spectra, along with the attenuation of the luminescence peak of BQ⊂CB[8]@SC4AD at 555 nm. As shown in Figure S21, Supporting Information, the emission of the nanosecond component of the phosphor would not diminish with the addition of Cy5, which indicated that the short‐lived fluorescent component did not participate in the process of energy transfer. The resultant phosphorescence peak at 675 nm was far beyond the initial emission at 555 nm in intensity. When the ratio of donor and receptor was 30:1, the phosphorescence spectral changes reached equilibrium state, indicating that there was an effective TS‐FRET taking place from triplet state of BQ⊂CB[8] to the singlet state of Cy5. The overall phosphorescence quantum yield for BQ⊂CB[8]@SC4AD:Cy5 system was measured to be 10.43% (Figure S22, Supporting Information). As expected, the phosphorescence lifetime at 555 nm of BQ⊂CB[8]@SC4AD:Cy5 system correspondingly shortened from 0.364 to 0.187 ms (Figure 3b). Meanwhile, this system exhibited a microsecond lifetime (0.160 ms) for this emerged red emissive peak at 675 nm (Figure S23, Supporting Information), which suggested that only by applying short wavelength (300 nm) to excite the BQ⊂CB[8]@SC4AD:Cy5 system, long‐lived emission from Cy5 could be monitored, further proofing the occurrence of TS‐FRET process from BQ⊂CB[8] to Cy5. In particular, both the luminescence peak of Cy5 in the BQ⊂CB[8]@SC4AD:Cy5 system and the fluorescence of Cy5 had the same maximum emission wavelength (675 nm) (Figure S24, Supporting Information), on behalf of the characteristic delayed‐fluorescence emission. The TS‐FRET efficiency can be calculated from the decrease of donor lifetime, and it was determined to be 48.6% under the donor/acceptor ratio of 30:1. These results indicated that through effectual TS‐FRET process, the long‐lived effective luminescence system BQ⊂CB[8]@SC4AD:Cy5 centered at 675 nm was successfully attained.

Given that the excellent long‐lived luminescence at 675 nm for BQ⊂CB[8]@SC4AD:Cy5 system, we further explored its possibility of constructing the secondary energy transfer system of cascaded FRET (TSS‐FRET) in aqueous solution. The establishment of phosphorescence harvesting system with long‐lived luminescence wavelength of more than 800 nm has so far not been reported in aqueous solution. IR780, a kind of the heptamethine carbocyanine dyes, exhibited a favorable NIR fluorescence emission (*λ*_em_ = 825 nm), which has been utilized as the NIR fluorescent probes for bioimaging^[^ ^27^ ^]^ and might be a suitable acceptor for the foundation of the long‐lived NIR emission system in aqueous solution. The absorption of IR780 and the emission of Cy5 show well spectral overlap to a certain extent (Figure 3e), which is a necessary condition to ensure the occurrence of energy transfer process (Figure S20, Supporting Information). As depicted in Figure 3c, with the addition of IR780, there was a clearly growing emission peak at 825 nm in the delayed spectra, accompanied by the substantial decreasing of the former peak at 675 nm as an indicative of the energy transfer from Cy5 to IR780. The global emission quantum yield for BQ⊂CB[8]@SC4AD:Cy5:IR780 was determined as 7.36% (Figure S25, Supporting Information). Moreover, after IR780 was directly added to the BQ⊂CB[8]@SC4AD assembly, there was almost no characteristic emission of IR780 in the region of 800–850 nm, and the phosphorescence emission band assigned to donor did not significantly reduce (Figure S26, Supporting Information), which ruled out the possibility that the donor effectively transferred energy to IR780. After introduction of IR780 into BQ⊂CB[8]@SC4AD:Cy5 system, the lifetime (675 nm) of BQ⊂CB[8]@SC4AD:Cy5:IR780 assigned to donor actually shortened to 85.0 µs from initial 0.160 ms (Figure 3d), further validating the result mentioned above. The lifetime at 555 nm for BQ⊂CB[8]@SC4AD:Cy5:IR780 was 0.185 ms (Figure S27, Supporting Information), which was similar to that of BQ⊂CB[8]@SC4AD:Cy5 system (0.187 ms). This new luminescent peak came from IR780 in the BQ⊂CB[8]@SC4AD:Cy5:IR780 system, whose location in delayed spectrum was in accordance with the fluorescent emission peak of IR780 indicative of typical delayed fluorescence (Figure S28, Supporting Information). Accordingly, it signified that the energy transfer came about from the singlet‐state of donor system to the singlet‐state of secondary acceptor IR780 (i.e., SS‐FRET). Besides, the lifetime at 825 nm for BQ⊂CB[8]@SC4AD:Cy5:IR780 system was 66.9 µs (Figure 3f), clearly demonstrating that the long‐lived fluorescence occurred through the delayed sensitization of the singlets of acceptor dye IR780 driven by the triplet‐state excitons‐involved energy transfer. IR780 luminescent peak for BQ⊂CB[8]@SC4AD:Cy5:IR780 system displayed the emission quenching phenomenon created by aggregation‐causing quenching (ACQ) effect with mounting inclusion content. In this process, the FRET efficiency for the SS‐FRET process was measured to be 46.9% according to the decrease of lifetime at 675 nm, in the circumstance of the virtually nonexistent energy transfer from BQ⊂CB[8] to IR780 directly. As a consequence, two different organic dyes could be successively co‐embedded into the BQ⊂CB[8]@SC4AD aggregates, and stepwise FRET processes would happen, first from the triplet‐state excitons of BQ⊂CB[8] to the singlet‐state excitons of Cy5, and then from the latter to the singlet‐state excitons of IR780, thus achieving the delayed sensitization for various organic dyes.

**Figure 4:**
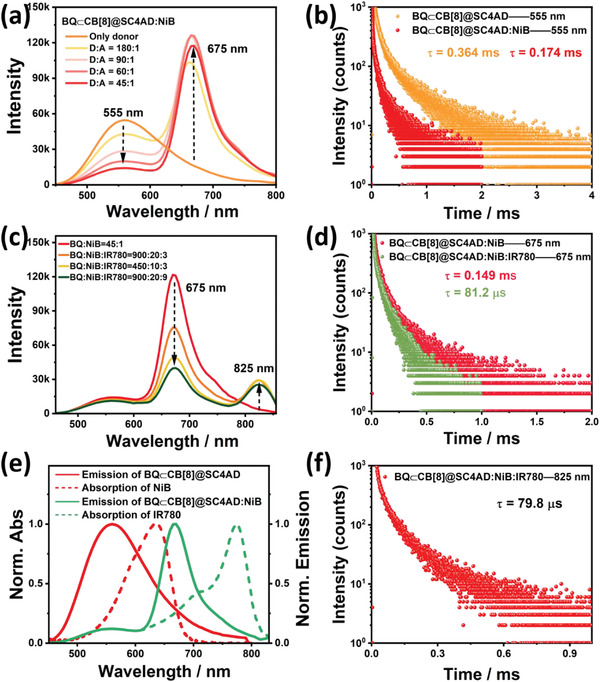
a) Phosphorescence emission spectra (delayed 50 µs) of BQ⊂CB[8]@SC4AD:NiB at different donor/acceptor ratios in aqueous solution at 298 K ([BQ⊂CB[8]] = 3.0 × 10^−5^ m, [SC4AD] = 1.5 × 10^−5^ m, *λ*_ex_ = 300 nm). b) Time‐resolved PL decay curves of BQ⊂CB[8]@SC4AD, BQ⊂CB[8]@SC4AD:NiB at 555 nm in aqueous solution at 298 K ([BQ⊂CB[8]] = 3.0 × 10^−5^ m, [SC4AD] = 1.5 × 10^−5^ m, [NiB] = 6.67 × 10^−7^ m). c) Phosphorescence emission spectra (delayed 50 µs) of BQ⊂CB[8]@SC4AD:NiB:IR780 at different donor/acceptor ratios in aqueous solution at 298K ([BQ⊂CB[8]] = 3.0 × 10^−5^ m, [SC4AD] = 1.5 × 10^−5^ m, [NiB] = 6.67 × 10^−7^ m, *λ*_ex_ = 300 nm). d) Time‐resolved PL decay curves of BQ⊂CB[8]@SC4AD:NiB, BQ⊂CB[8]@SC4AD:NiB:IR780 at 675 nm in aqueous solution at 298 K ([BQ⊂CB[8]] = 3.0 × 10^−5^ m, [SC4AD] = 1.5 × 10^−5^ m, [NiB] = 6.67 × 10^−7^ m, [IR780] = 3.0 × 10^−7^ m). e) Normalized emission spectrum of BQ⊂CB[8]@SC4AD (red solid line) and BQ⊂CB[8]@SC4AD:NiB (green solid line) assembly and absorption spectra of NiB (red short dash) and IR780(green short dash). f) Time‐resolved PL decay curves of BQ⊂CB[8]@SC4AD:NiB:IR780 at 825 nm in aqueous solution at 298 K ([BQ⊂CB[8]] = 3.0 × 10^−5^ m, [SC4AD] = 1.5 × 10^−5^ m, [NiB] = 6.67 × 10^−7^ m, [IR780] = 3.0 × 10^−7^ m).

**Figure 5:**
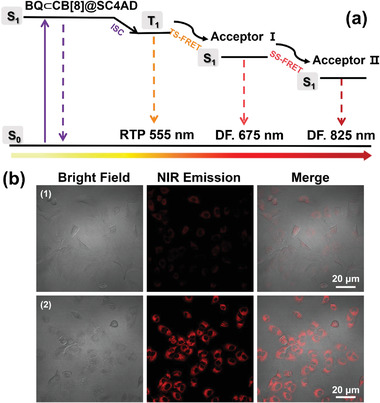
a) Simplified Jablonski diagram to illustrate the probable mechanism for stepwise phosphorescence harvesting process. (ISC = intersystem crossing, TS‐FRET = Triplet to singlet Förster resonance energy transfer, SS‐FRET = Singlet to singlet Förster resonance energy transfer. b) Confocal microscopy images of A549 cells costained with IR780 (1), and BQ⊂CB[8]@SC4AD:Cy5:IR780 (2) (Left: bright field; middle: *λ*_ex_ = 405 nm, *λ*_em_ = 650–800 nm; right: merged [BQ⊂CB[8]] = 3.0 × 10^−5^ m, [SC4AD] = 3.0 × 10^−5^ m, [Cy5] = 1.0 × 10^−6^ m, [IR780] = 3.33 × 10^−7^ m).

To validate the adaptability of stepwise phosphorescence artificial harvesting in BQ⊂CB[8]@SC4AD system with IR780 as the final acceptor, we found out another intermediate acceptor dye, NiB whose absorption and emission region can feasibly cover with the emission band of BQ⊂CB[8]@SC4AD system and the absorption region of IR780, individually (**Figure** **4**e and Figure S20, Supporting Information). As shown in Figure 4a, as introduction of NiB into the BQ⊂CB[8]@SC4AD system, there was a gradual escalation of an additional peak centered at 675 nm, coming with the decrease of phosphorescence emission intensity of BQ⊂CB[8]@SC4AD at 555 nm. Steady‐state emission spectrum experiments were also carried out to study this process (Figure S29, Supporting Information). NiB would suffer from emission quenching when the ratio of BQ⊂CB[8]@SC4AD:NiB exceeded 60:1 due to the ACQ effect, and its location was consistent with the fluorescent emission peak of NiB (Figure S30, Supporting Information). At the same time, the donor lifetime shortened from 0.364 to 0.174 ms (Figure 4b), which brought forth the conclusion of a TS‐FRET efficiency of 52.2%. Besides, the lifetime of NiB for BQ⊂CB[8]@SC4AD:NiB system was determined as 0.149 ms according to the time‐resolved PL decay curve analyses (Figure S31, Supporting Information). Interestingly, after doping IR780 into BQ⊂CB[8]@SC4AD:NiB system, a new peak belonging to IR780 was discovered in the region of 800–850 nm except the decreased emission peak at 675 nm, which owned the same maximum wavelength with the fluorescence of IR780 (Figure 4c and Figures S32 and S33, Supporting Information). During this process, the lifetime at 675 nm for BQ⊂CB[8]@SC4AD:NiB:IR780 lessened to 81.2 µs (Figure 4e), and it was determined to be microsecond level at 825 nm (79.8 µs) (Figure 4f). The SS‐FRET efficiency was calculated as 45.5% according to the decrease of lifetime. In addition, the lifetime collected at 555 nm for BQ⊂CB[8]@SC4AD:NiB:IR780 was 0.173 ms (Figure S34, Supporting Information). The phosphorescence quantum yield for BQ⊂CB[8]@SC4AD:NiB was 6.01% (Figure S35, Supporting Information), and was 5.83% for BQ⊂CB[8]@SC4AD:NiB:IR780 (Figure S36, Supporting Information). Conjointly, NiB also could function as a bridge to realize cascaded energy transfer from donor to first acceptor then to final acceptor (IR780) to achieve the ultralarge Stokes shift and long‐lived NIR (825 nm) emission in aqueous solution. And most remarkably, two‐step energy transfer process of TS‐FRST and SS‐FRET had access to achievement of delayed sensitization for primary and secondary receptors, and the probable mechanism of stepwise phosphorescence harvesting system is displayed in **Figure** **5**a.

The remarkable phosphorescence‐harvesting behavior of BQ⊂CB[8]@SC4AD:Cy5:IR780 prompted us to investigate whether it can be used as an imaging agent for NIR cell labeling. We initially examined the cytotoxicity of this system toward human lung adenocarcinoma cells as model (A549 cancer cells). The results from standard cell counting kit‐8 analysis revealed that above system presented negligible toxicity, and the cell survival rate remained above 90%, even at very high concentrations 50 µm (Figure S37, Supporting Information). Next, confocal laser scanning microscopy was performed to study its feasibility as NIR cell labeling agent. As shown in Figure 5b, it could be seen that cells treated with BQ⊂CB[8]@SC4AD:Cy5:IR780 system emitted the bright red luminescence signal, while for the free heptamethine carbocyanine dyes, IR780, there was a negligible emission signal in red channel. To further investigate the localization of the obtained assembly in cell organelles, colocalization experiments were carried out. It turned out that the NIR emission of BQ⊂CB[8]@SC4AD:Cy5:IR780 system in the red channel showed considerable overlap with the MitoTracker Green emission in the green channel, and the Pearson correlation coefficient was as high as 0.89, suggesting that it mainly distributed in mitochondria (Figure S38, Supporting Information). The above experimental results demonstrated that the obtained phosphorescent artificial harvesting system BQ⊂CB[8]@SC4AD:Cy5:IR780 can act as a promising NIR cell labeling imaging agent with low toxicity.

## 3 Conclusion

In conclusion, a stepwise artificial phosphorescence harvesting system with ultralarge Stokes shift (≈525 nm) and NIR emission at 825 nm was successfully constructed via the cascaded supramolecular assembly of BQ, CB[8], and SC4AD, which was successfully applied to NIR cell labeling. Taking advantage of the synergistic effect of CB[8] macrocycle‐confined phosphorescence emission and assembling‐enhanced emission, the resultant cascaded supramolecular assembly BQ⊂CB[8]@SC4AD exhibited desired phosphorescence performance at 555 nm, which further served as a versatile light‐harvesting platform to build efficient two‐step sequential phosphorescence energy transfer via successively introducing the primary acceptors (Cy5 or NiB) and secondary acceptors (IR780). Specifically, TS‐FRET process took place from BQ⊂CB[8]@SC4AD aggregate to the intermediate acceptor (Cy5, NiB), and then to the final NIR dye (IR780) through SS‐FRET process. This stepwise phosphorescence energy transfer system possessing long lifetime and long wavelength behaviors provides a convenient and viable method for creation of long‐lived NIR luminescence materials and has great potential application in bioimaging.

## Conflict of Interest

The authors declare no conflict of interest.

## Supporting information

Supporting Information

## Data Availability

The data that support the findings of this study are available in the Supporting Information of this article.
