# Supplementary material for: Ultralarge Stokes Shift Phosphorescence Artificial Harvesting Supramolecular System with Near‐Infrared Emission
Source: Adv Sci (Weinh). 2022 Jun 2;9(22):2201523. doi: 10.1002/advs.202201523 (PMC9353443; doi:10.1002/advs.202201523)
Supplement: Supplementary file 1 — Supporting Information [file ADVS-9-2201523-s001.pdf]

ADVANCED SCIENCE
Open Access

Supporting Information

for *Adv. Sci.*, DOI 10.1002/advs.202201523

Ultralarge Stokes Shift Phosphorescence Artificial Harvesting Supramolecular System with Near-Infrared Emission

*Man Huo, Xian-Yin Dai and Yu Liu**

Supporting Information

Ultralarge Stokes Shift Phosphorescence Artificial Harvesting Supramolecular System with Near-Infrared Emission

*Man Huo, Xian-Yin Dai, and Yu Liu**

Dr. M. Huo, Dr. X.-Y. Dai, Prof. Y. Liu

College of Chemistry, State Key Laboratory of Elemento-Organic Chemistry, Nankai University, Tianjin 300071, P. R. China

E-mail: yuliu@nankai.edu.cn

Table of Contents

Section A. Instrumentation and Methods….S1

Section B. Synthetic Protocols…..S2

Section C. Host-Guest Properties of BQ and CB[8]…..S5

Section D. Characterizations of BQ⊂CB[8]@SC4AD…..S10

Section E. Characterizations of Phosphorescence Energy Transfer Behaviors of BQ⊂CB[8]@SC4AD…..S13

Section F. References…..S24

Section A. Instrumentation and Methods

All reagents and solvents were obtained from commercial suppliers and were used as supplied, unless otherwise noted. NMR spectra were recorded on an Ascend 400 MHz instrument. High-resolution MS was performed on a Q-TOF LC-MS in electrospray ionization mode. UV-vis absorption spectra were recorded on a Shimadzu UV-3600 spectrophotometer with a PTC-348WI temperature controller in a quartz cell (light path 10 mm) at 298 K. Photoluminescence spectra, lifetime and quantum efficiency were obtained on FLS980 instrument (Edinburg Instruments, Livingstone, UK). The photoluminescence spectra for each data point was delayed 50 μs to ensure that the first 50 μs are not collected to eliminate the fluorescence emission. TEM experiments were carried out on a FEI Tecnai G2 F20 microscope operating at 200 KV and the samples were prepared by dropping the solution onto a copper grid which were then air-dried. Scanning electron microscopy images were obtained with a Hitachi S-3500 N scanning electron microscope. Dynamic Light Scattering (DLS) was examined by using a laser light scattering spectrometer (BI-200SM) equipped with a digital correlator (Turbo Corr) at 636 nm at a scattering angle of 90°. The hydrodynamic diameter (Dh) was determined by DLS experiments at 298 K. The confocal images were carried out on Olympus FV1000 confocal laser scanning microscope. Time-resolved fluorescence (TRF) and CCK8 assay were detected by Microplate Reader setting with 50 microsecond delay time (American BioTek Synergy 4).

Cell viability assay.

Human lung cancer cell line A549 cells were purchased from Cell Resource Center, Chinese Academy of Medical Science Beijing. A549 cells were incubated by using Ham's F12 nutrient medium supplemented with 10 % FBS and 1 % penicillin/streptomycin in a humidified incubator with 5% CO_2 atmosphere at 37 ℃. A549 cells were seeded in 96-well plates for 24 h at 37 ℃ in 5% CO_2. Then the cells were incubated with **BQ**⊂CB[8]@SC4AD: Cy5:IR780 at different concentrations for 24 h. The relative cellular viability was determined by the CCK8 assay.

Confocal laser scanning microscopy.

A549 cells were seeded in o a confocal petri dish for 24 h at 37 ℃ in 5% CO_2. The cells were

incubated with the **BQ**⊂CB[8]@SC4AD: Cy5:IR780 for 12 h. Then the medium was removed, and the cells were washed with phosphate buffer solution for three time. After the cells were repeatedly washed at least three times with PBS, the cells were subjected to observation by CLSM.

Statistical Analysis.

Three replicates of each experiment were performed, and values herein stand for means ± standard deviations (SD). Differences between groups were compared by a one-way analysis of variance test ($p < 0.05$). All statistical tests were performed using the SPSS software package (ver. 20, IBM, USA).

Section B. Synthetic Protocols

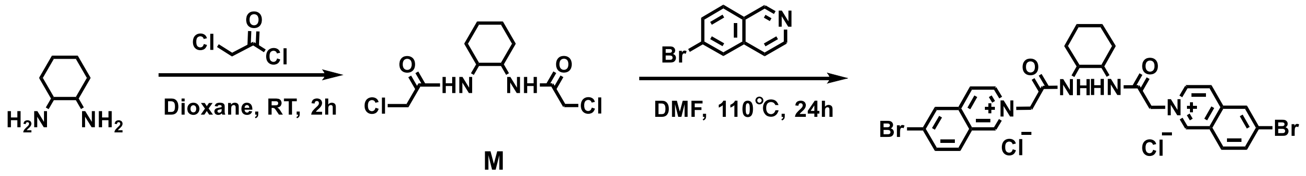

Scheme S1. Synthetic routes of **BQ**.

Compound **M**: Compound **M** was synthesized according to the previous report.[1]

Compound **BQ**: Compound **M** (0.267 g, 1mmol) and 6-bromoisoquinoline (0.499 g, 2.4 mmol) were added in 10 mL of anhydrous N,N'-dimethylformamide solution. After reacting at 100 °C in oil bath for 48 h, a large amount of precipitate was deposited. After cooling, the solid was collected by filtration, washed with 50 mL of dichloromethane, 100 mL of ether, and dried to obtain the product (0.389 g, 56.9%). ^{1}H NMR (400 MHz, DMSO-d_6) δ 10.32 (s, 2H), 8.92 (d, J = 6.8 Hz, 2H), 8.74 (dd, J = 12.4, 4.1 Hz, 4H), 8.49 (dd, J = 13.3, 7.9 Hz, 4H), 8.24 (dd, J = 8.9, 1.9 Hz, 2H), 5.85 (d, J = 15.7 Hz, 2H), 5.53 (d, J = 15.8 Hz, 2H), 3.64 (s, 2H), 1.88 (d, J = 12.6 Hz, 2H), 1.68 (s, 2H), 1.38 (s, 2H), 1.26 (t, J = 9.6 Hz, 2H). ^{13}C NMR (100 MHz, DMSO-d_6) δ 164.52, 152.40, 138.39, 138.07, 135.01, 132.73, 132.51, 130.17, 125.94, 124.43, 61.93, 52.63, 31.72, 24.43, 0.58. HRMS (ESI) m/z for $C_{28}H_{28}Br_2Cl_2N_4O_2$ calcd. $[M{-}2Cl]^{2+}$: 306.0274, found: 306.0271.

IR780: IR780 was prepared according to the literature[2].

SC4AD: SC4AD was prepared according to the literature[3].

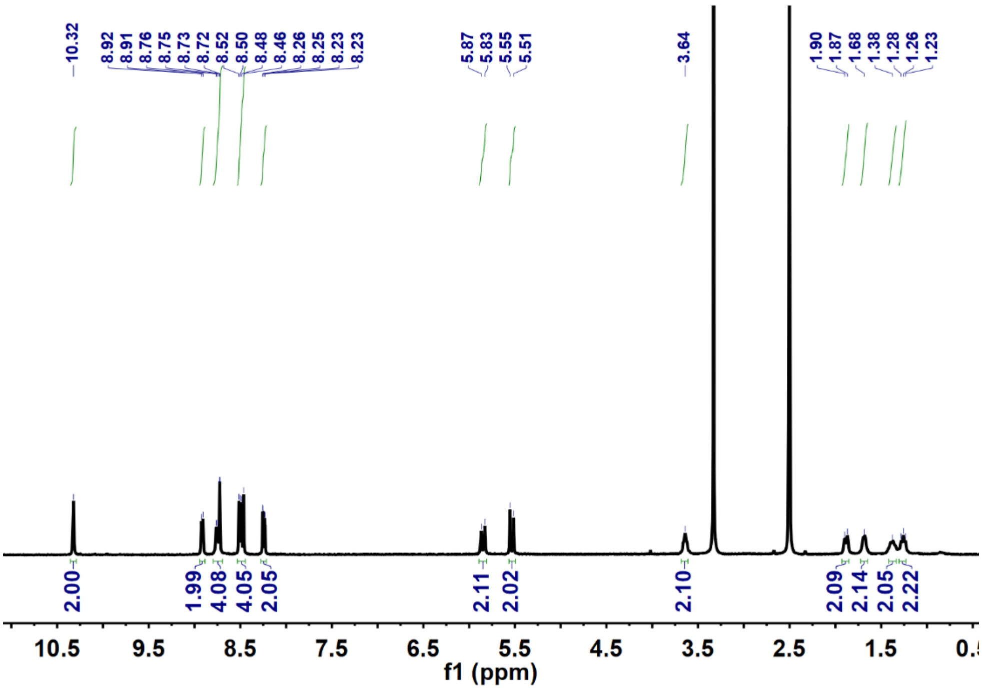

Figure S1. ^{1}H NMR spectrum (400 MHz, DMSO-d_6, 298 K) of **BQ**.

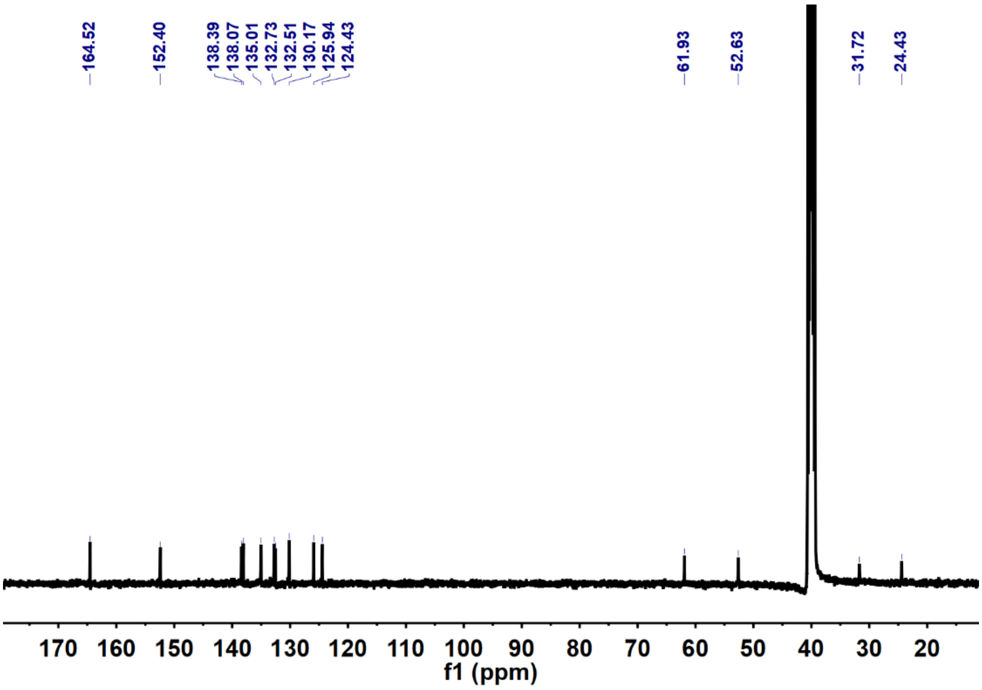

Figure S2. ^{1}C NMR spectrum (100 MHz, DMSO-d_6, 298 K) of **BQ**.

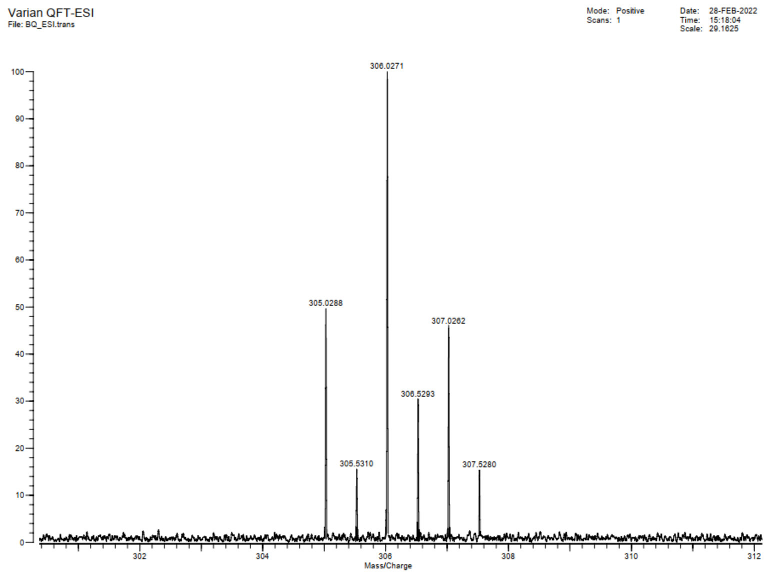

Figure S3. HR-MS spectrum of **BQ**.

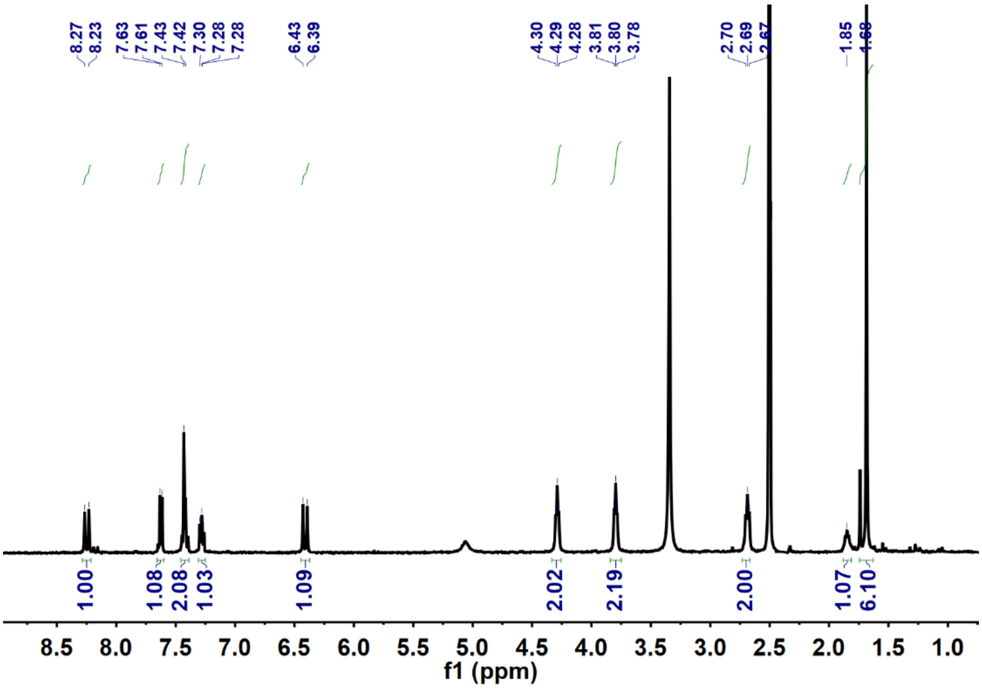

Figure S4. ^{1}H NMR spectrum (400 MHz, DMSO-d_6, 298 K) of IR780.

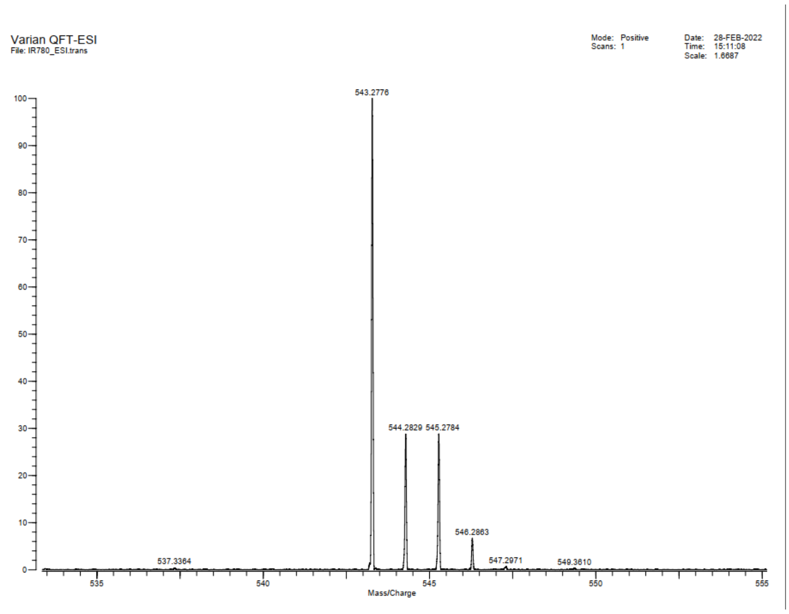

Figure S5. HR-MS spectrum of IR780.

Section C. Host-Guest Properties of BQ and CB[8]

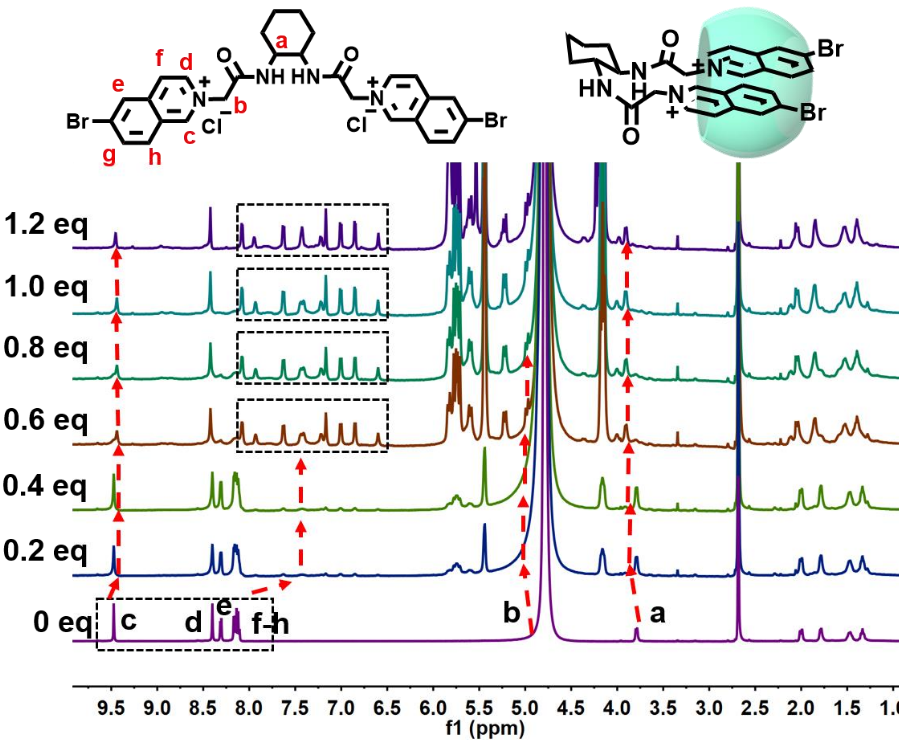

Figure S6. Changes of the ^{1}H NMR signals of **BQ** (600 MHz, D_2O, 298K) at different ratios of **BQ** and CB[8] ([**BQ**] = 2.0 × 10^{-4} M, [CB[8]] = 0−2.4 × 10^{-4} M).

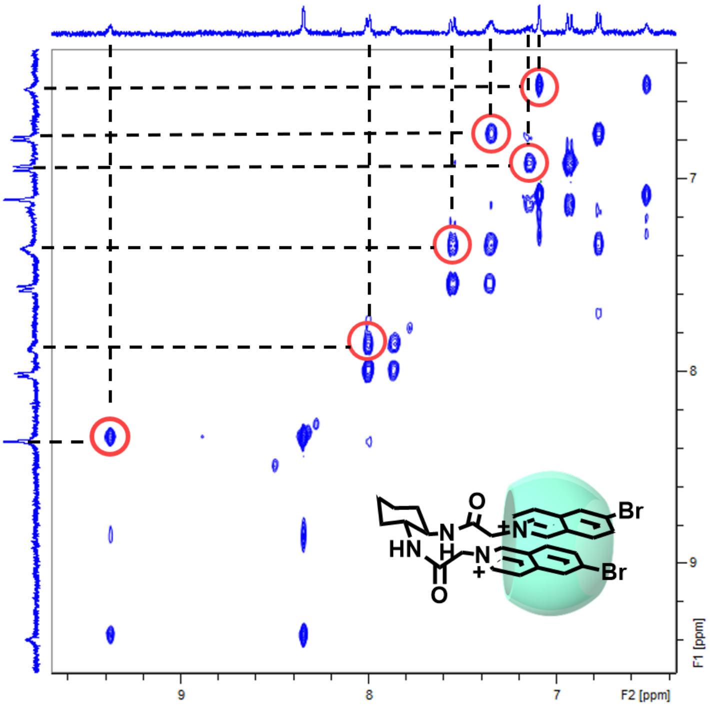

Figure S7. ROESY spectra of **BQ**⊂CB[8] ([**BQ**] = 2.0 × 10^{-4} M, [CB[8]] = 2.0 × 10^{-4} M) in D_2O at 25 °C.

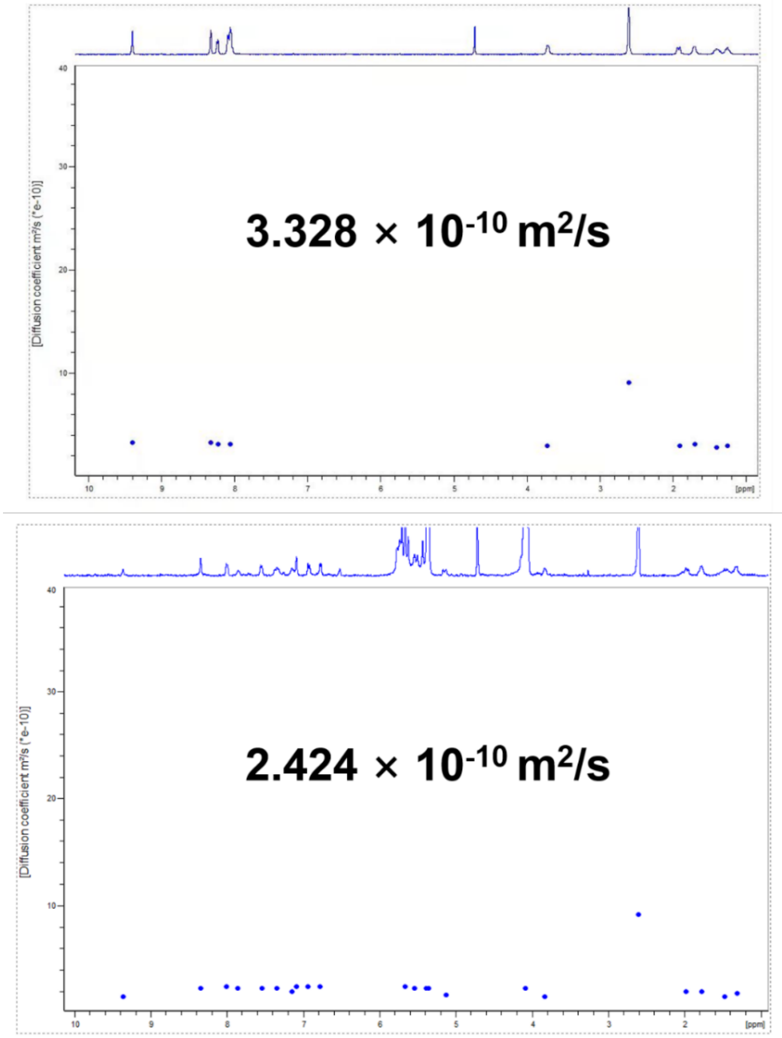

Figure S8. 2D DOSY spectra (400 MHz, D_2O, 298 K). (a): Diffusion coefficient of **BQ**. (b): Diffusion coefficient of **BQ**⊂CB[8] ([**BQ**] = 2.0 × 10^{-4} M, [CB[8]] = 2.0 × 10^{-4} M).

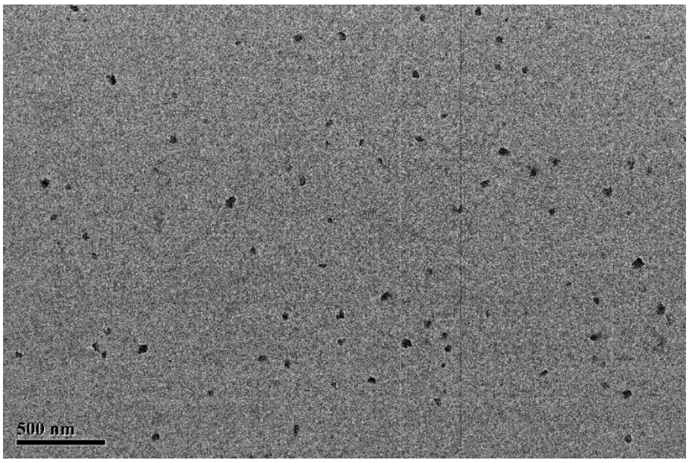

Figure S9. (a) TEM image of **BQ**⊂CB[8] ([**BQ**] = 1.0 × 10^{-5} M, [CB[8]] = 1.0 × 10^{-5} M).

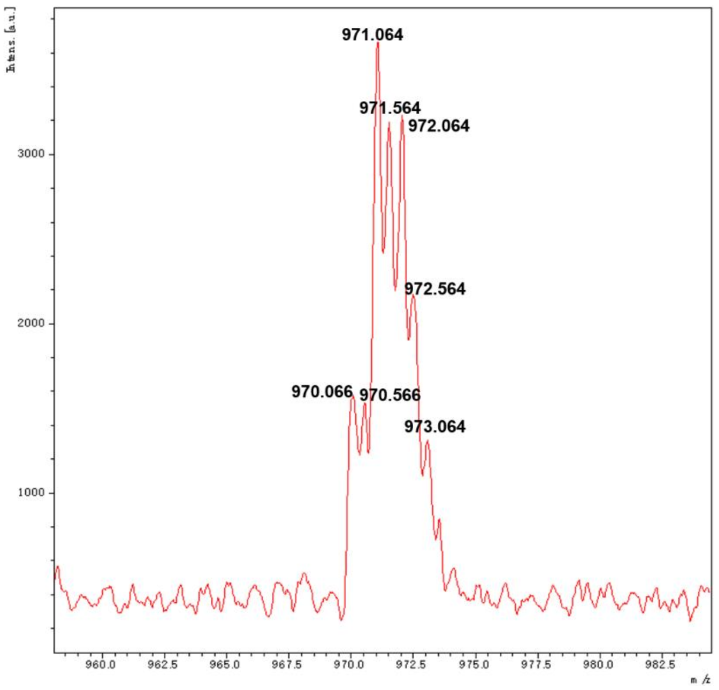

Figure S10. MALDI-TOF-HRMS spectrum of **BQ**⊂CB[8].

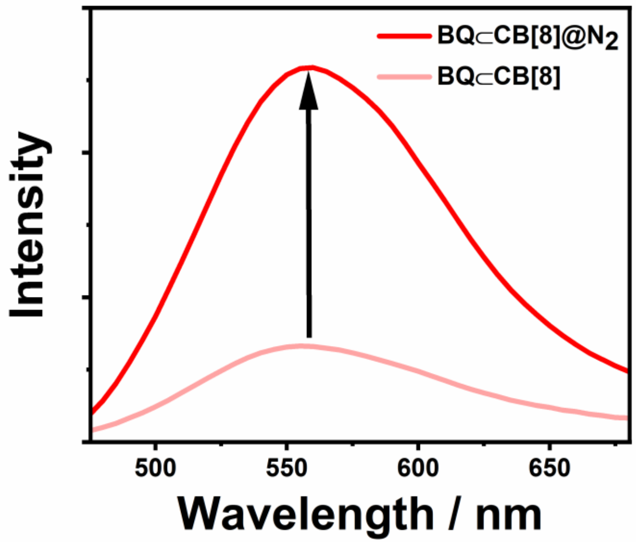

Figure S11. Phosphorescence emission spectra (delayed 50 μs) of **BQ**⊂CB[8], **BQ**⊂CB[8]@N_2, (λ_{ex} = 300 nm) in aqueous solution at 298 K ([**BQ**] = 1.0 × 10^{-5} M, [CB[8]] = 1.0 × 10^{-5} M).

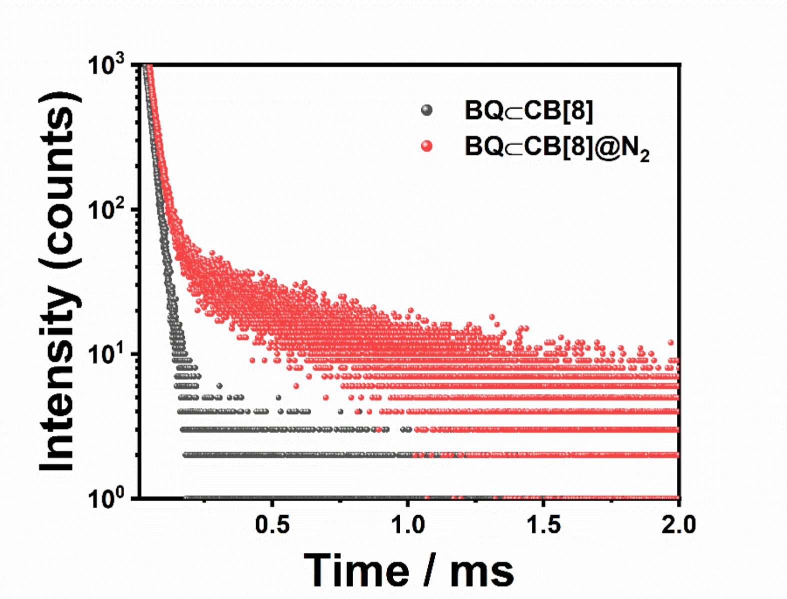

Figure S12. The time-resolved PL decay spectrum of **BQ**⊂CB[8] before and after vacuum degassing in aqueous solution at 298 K (λ_{ex} = 300 nm) ([**BQ**] = 1.0 × 10^{-5} M, [CB[8]] = 1.0 × 10^{-5} M).

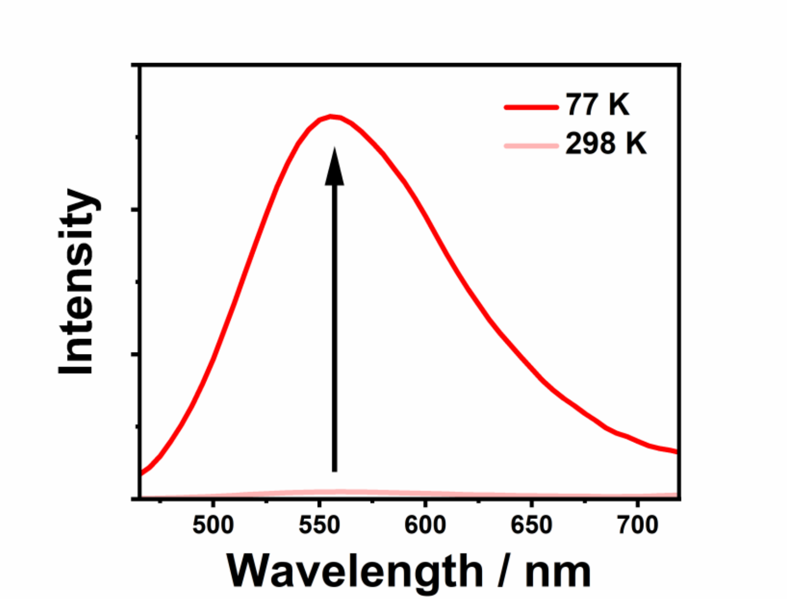

Figure S13. Phosphorescence emission spectra (delayed 50 µs) of **BQ**⊂CB[8] (λ_{ex} = 300 nm) in aqueous solution at 77 K and at 298 K ([**BQ**] = 1.0 × 10^{-5} M, [CB[8]] = 1.0 × 10^{-5} M).

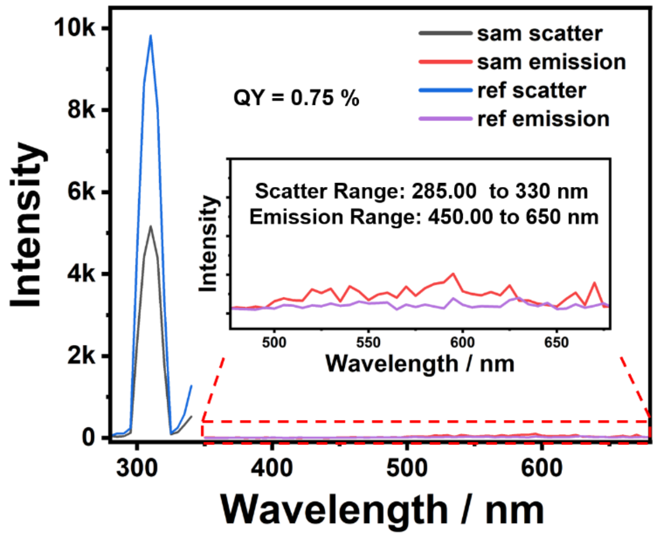

Figure S14. The phosphorescence quantum yield of **BQ**⊂CB[8] under ambient conditions.

Section D. Characterizations of BQ⊂CB[8]@SC4AD

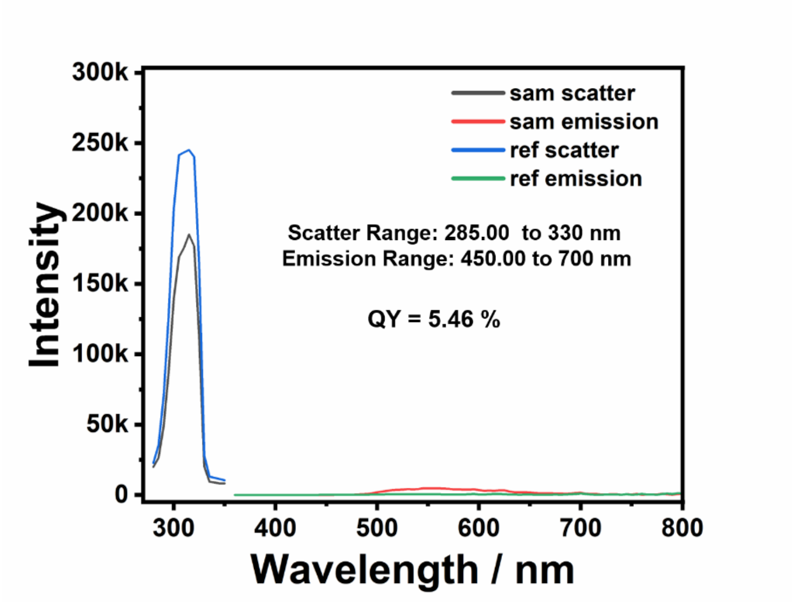

Figure S15. The phosphorescence quantum yield of **BQ**⊂CB[8]@SC4AD under ambient conditions.

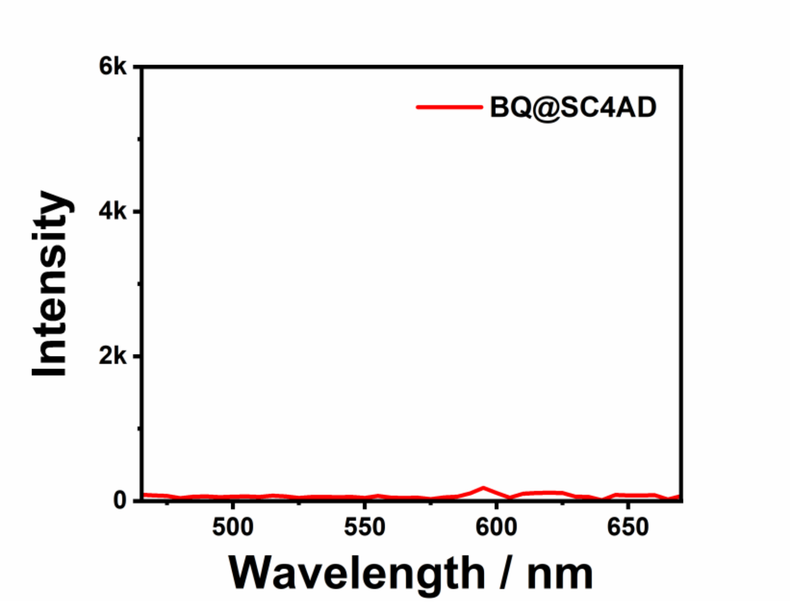

Figure S16. Phosphorescence emission spectra (delayed 50 μs) of **BQ**@SC4AD (λ_{ex} = 300 nm) in aqueous solution at 298 K ([**BQ**] = 3.0 × 10^{-5} M, [SC4AD] = 1.5 × 10^{-5} M).

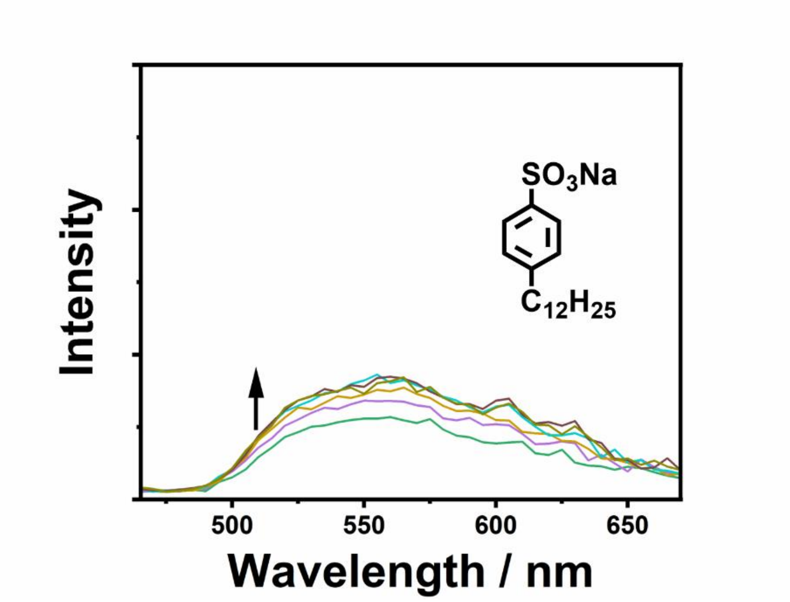

Figure S17. Effect of the addition of SDBS (0–1.5 × 10^{-5} M) on the phosphorescence emission spectra of **BQ**@CB[8] (3.0 × 10^{-5} M) in aqueous solution at 298 K (λ_{ex} = 300 nm).

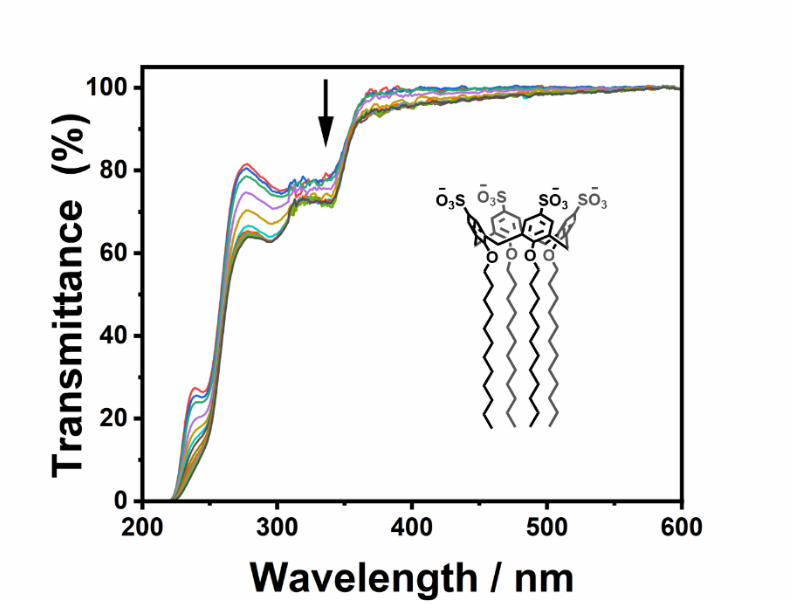

Figure S18. Transmittance changes of **BQ**⊂CB[8] in varying concentration of SC4AD from 0 to 2.0 × 10^{-5} M ([**BQ**] = [CB[8]] = 3.0 × 10^{-5} M), respectively.

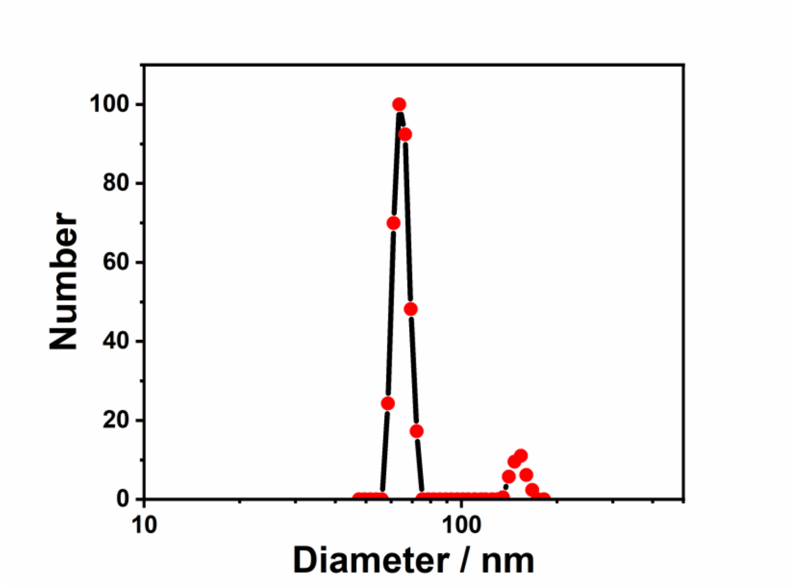

Figure S19. DLS data of **BQ**⊂CB[8]@SC4AD ternary assembly ([**BQ**] = 1.0 × 10^{-5} M, [CB[8]] = 1.0 × 10^{-5} M, [SC4AD]= 5 × 10^{-6} M).

Section E. Characterizations of phosphorescence energy transfer behaviors of BQ⊂CB[8]@SC4AD

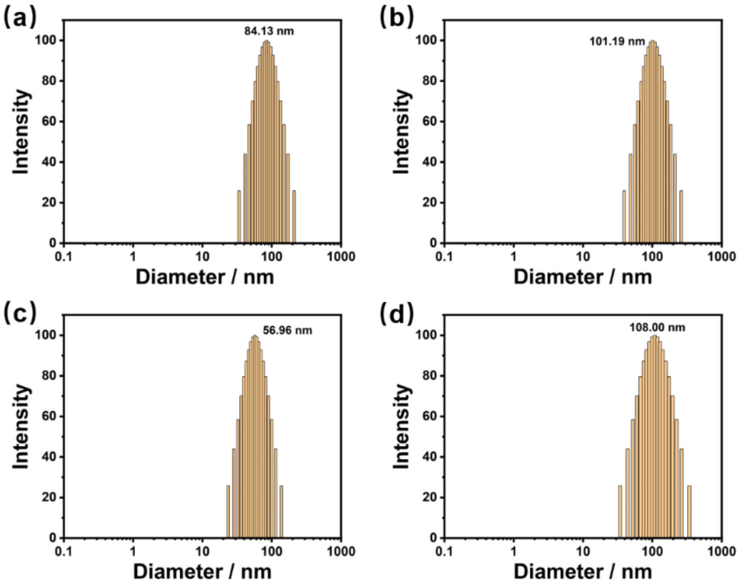

Figure S20. DLS data of a) **BQ**⊂CB[8]@SC4AD:Cy5 assembly, b) **BQ**⊂CB[8]@SC4AD:Cy5:IR780 assembly, c) **BQ**⊂CB[8]@SC4AD:NiB assembly and d) **BQ**⊂CB[8]@SC4AD:NiB:IR780 assembly in water at 25 °C.

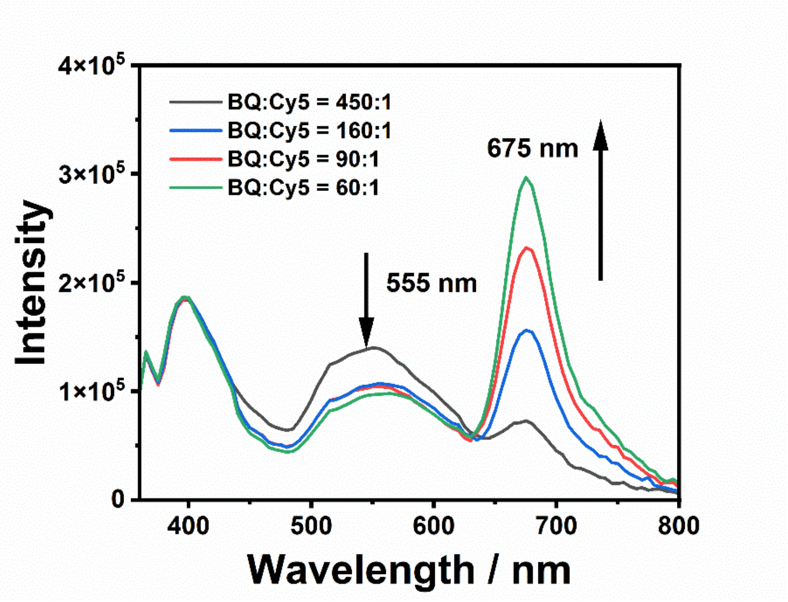

Figure S21. Steady-state emission spectra of **BQ**⊂CB[8]@SC4AD:Cy5 at different donor/acceptor ratios in aqueous solution at 298 K ([**BQ**⊂CB[8]] = 3.0×10^{-5} M, [SC4AD] = 1.5×10^{-5} M, λ_{ex} = 300 nm).

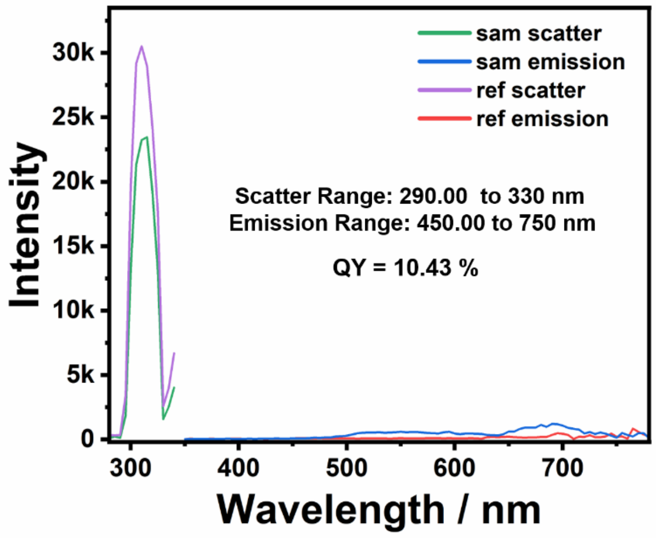

Figure S22. The phosphorescence quantum yield of **BQ**⊂CB[8]@SC4AD:Cy5 under ambient conditions.

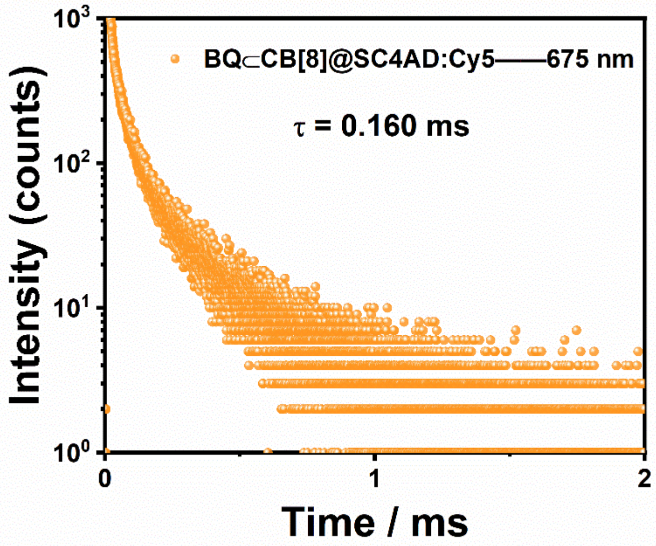

Figure S23. The time-resolved PL decay spectrum of **BQ**⊂CB[8]@SC4AD:Cy5 at 675 nm (λ_{ex} = 300 nm) ([SC4AD] = 1.5 × 10^{-5} M, [**BQ**] = 3.0 × 10^{-5} M, [CB[8]] = 3.0 × 10^{-5} M, [Cy5] = 1.0 × 10^{-6} M).

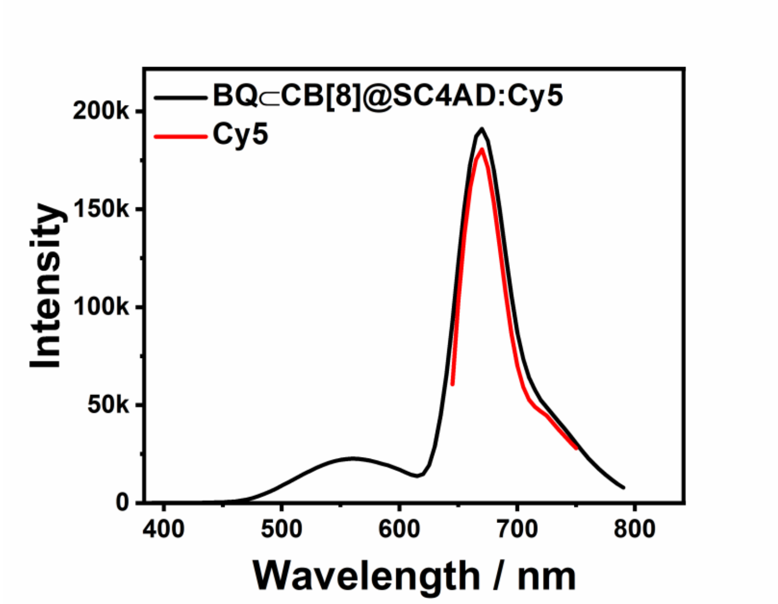

Figure S24. Normalized phosphorescence emission spectrum (delayed 50 μs) of **BQ**⊂CB[8]@SC4AD:Cy5 ([Cy5] = 1.0 × 10^{-6} M) and fluorescence emission spectrum of Cy5 ([Cy5] = 1.0 × 10^{-5} M) in aqueous solution (λ_{ex} = 300 nm, 298 K). ([SC4AD] = 1.5 × 10^{-5} M, [**BQ**] = 3.0 × 10^{-5} M, [CB[8]] = 3.0 × 10^{-5} M).

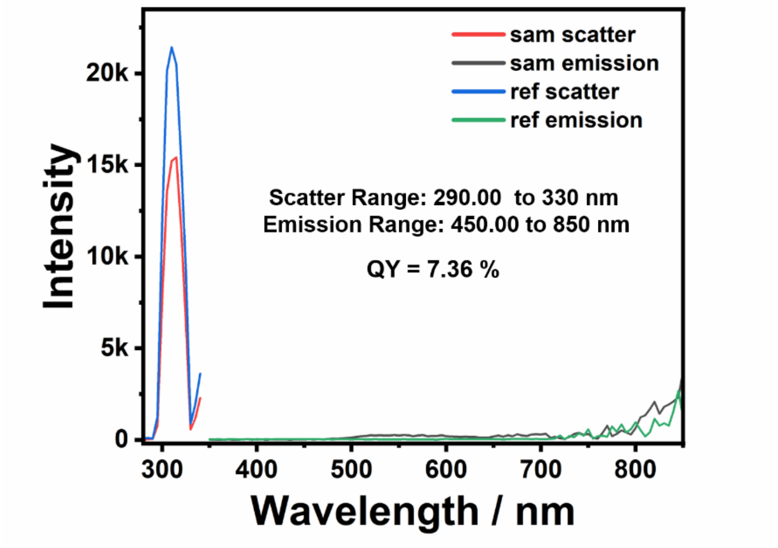

Figure S25. The phosphorescence quantum yield of **BQ**⊂CB[8]@SC4AD:Cy5:IR780 under ambient conditions.

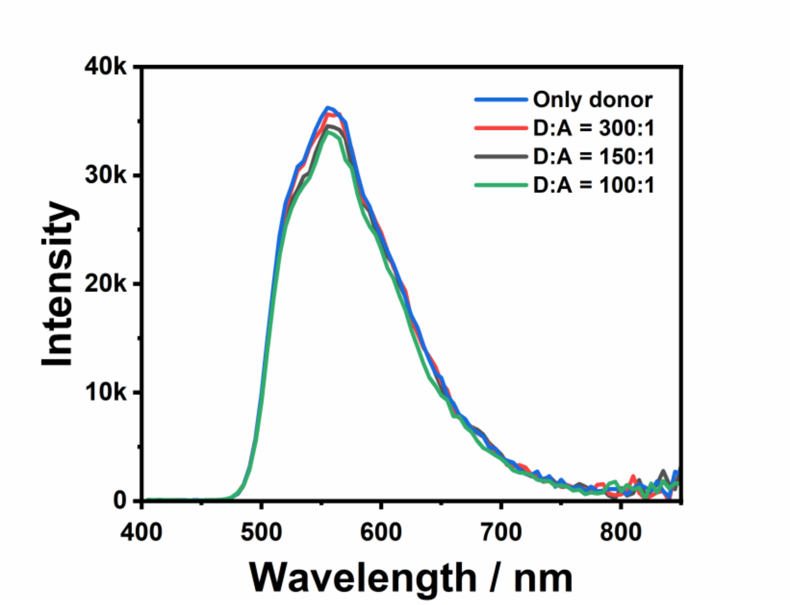

Figure S26. Phosphorescence emission spectra (delayed 50 μs) of **BQ**⊂CB[8]@SC4AD:IR780 at different donor/acceptor ratios in aqueous solution at 298 K.

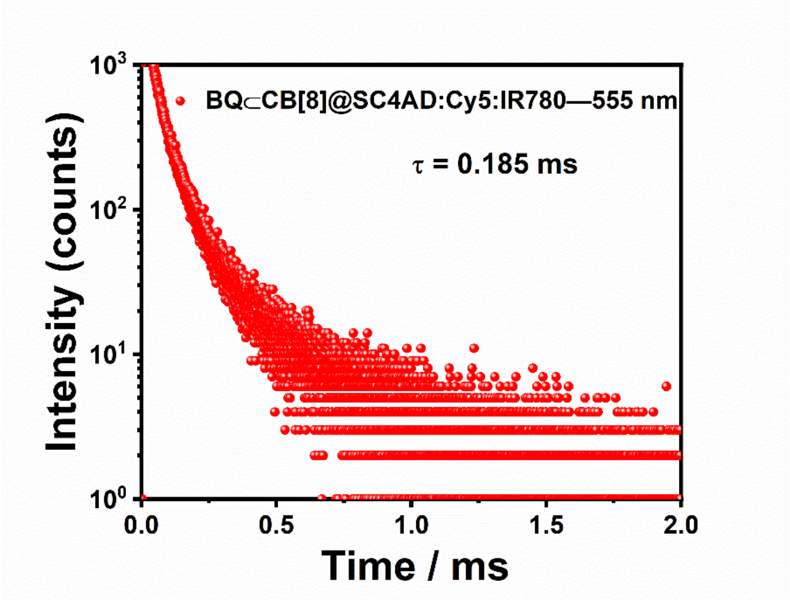

Figure S27. Time-resolved PL decay curves of **BQ**⊂CB[8]@SC4AD:Cy5:IR780 at 555 nm in aqueous solution at 298 K ([**BQ**⊂CB[8]] = 3.0×10^{-5} M, [SC4AD] = 1.5×10^{-5} M, [Cy5] = 1.0×10^{-6} M, [IR780] = 3.33×10^{-7} M).

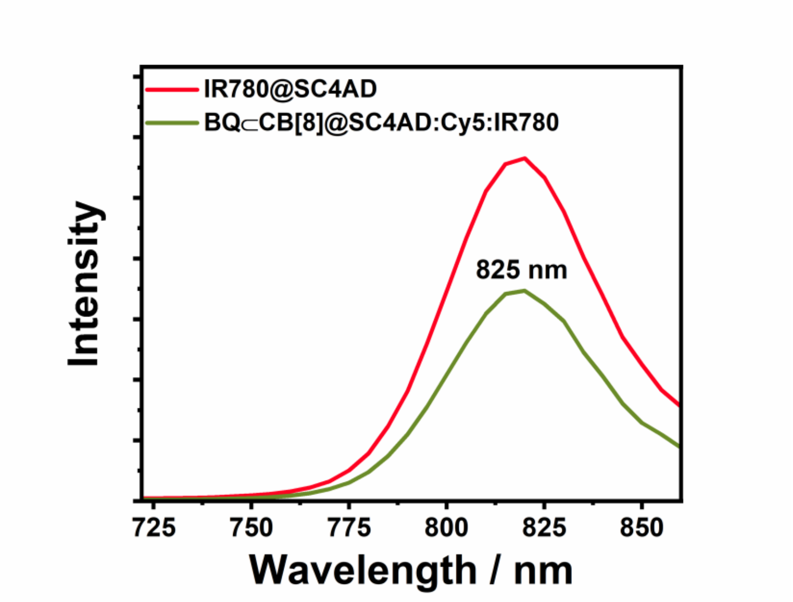

Figure S28. Normalized phosphorescence emission spectrum (delayed 50 μs) of **BQ**⊂CB[8]@SC4AD:Cy5:IR780 ([Cy5] = 1.0 × 10^{-6} M) and fluorescence emission spectrum of IR780 ([IR780] = 1.0 × 10^{-5} M) in aqueous solution (λ_{ex} = 300 nm, 298 K). ([SC4AD] = 1.5 × 10^{-5} M, [**BQ**] = 3.0 × 10^{-5} M, [CB[8]] = 3.0 × 10^{-5} M).

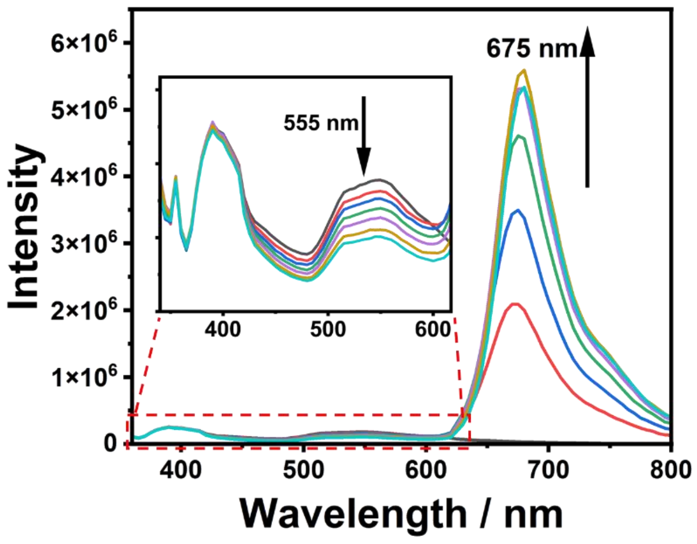

Figure S29. Steady-state emission spectra of **BQ**⊂CB[8]@SC4AD:NiB at different donor/acceptor ratios in aqueous solution at 298 K ([**BQ**⊂CB[8]] = 3.0 × 10^{-5} M, [SC4AD] = 1.5 × 10^{-5} M, λ_{ex} = 300 nm).

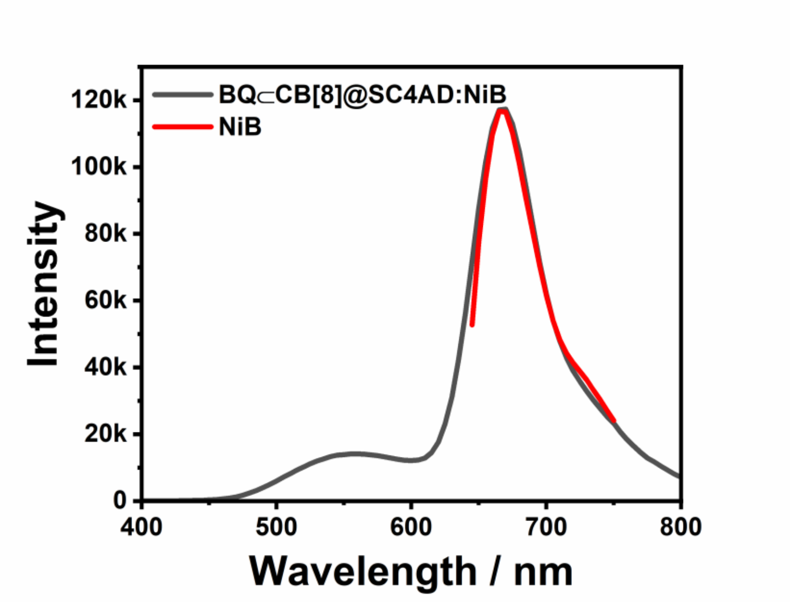

Figure S30. Normalized phosphorescence emission spectrum (delayed 50 μs) of **BQ**⊂CB[8]@SC4AD:NiB ([NiB] = 6.67 × 10^{-7} M) and fluorescence emission spectrum of NiB ([NiB] = 1.0 × 10^{-5} M) in aqueous solution (λ_{ex} = 300 nm, 298 K). ([SC4AD] = 1.5 × 10^{-5} M, [**BQ**] = 3.0 × 10^{-5} M, [CB[8]] = 3.0 × 10^{-5} M).

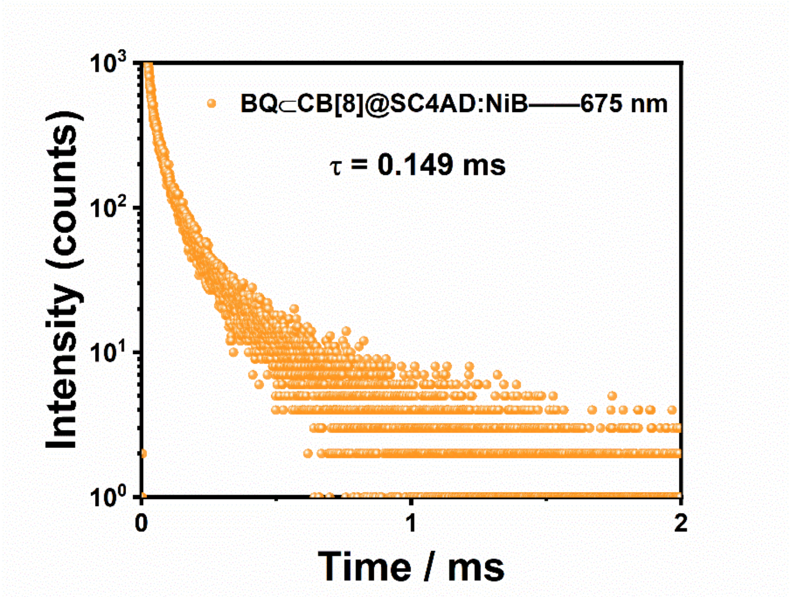

Figure S31. The time-resolved PL decay spectrum of **BQ**⊂CB[8]@SC4AD:NiB at 675 nm (λ_{ex} = 300 nm) ([SC4AD] = 1.5 × 10^{-5} M, [**BQ**] = 3.0 × 10^{-5} M, [CB[8]] = 3.0 × 10^{-5} M, [NiB] = 6.67 × 10^{-7} M).

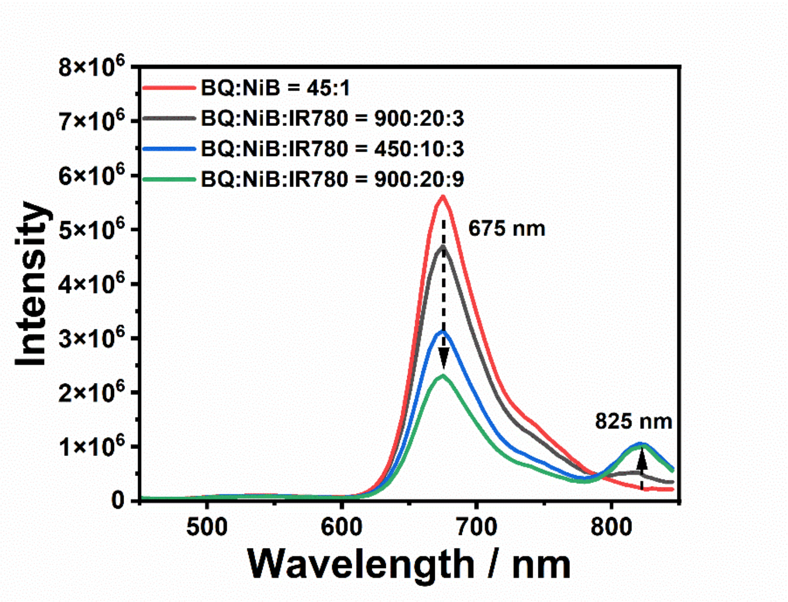

Figure S32. Steady-state emission spectra of of **BQ**⊂CB[8]@SC4AD:NiB:IR780 at different donor/acceptor ratios in aqueous solution at 298K ([**BQ**⊂CB[8]] = 3.0 × 10^{-5} M, [SC4AD] = 1.5 × 10^{-5} M, [NiB] = 6.67 × 10^{-7} M, λ_{ex} = 300 nm).

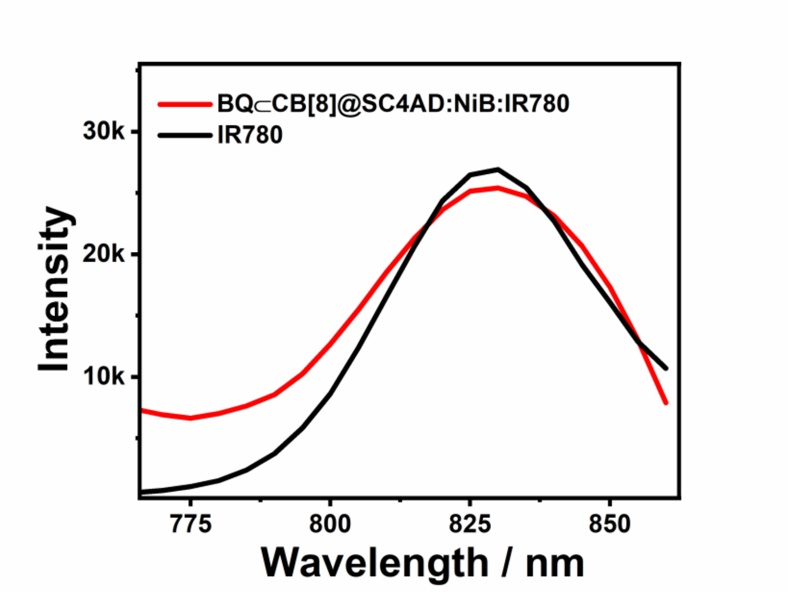

Figure S33. Normalized phosphorescence emission spectrum (delayed 50 μs) of **BQ**⊂CB[8]@SC4AD:NiB:IR780 ([NiB] = 6.67 × 10^{-7} M) and fluorescence emission spectrum of IR780 ([IR780] = 1.0 × 10^{-5} M) in aqueous solution (λ_{ex} = 300 nm, 298 K). ([SC4AD] = 1.5 × 10^{-5} M, [**BQ**] = 3.0 × 10^{-5} M, [CB[8]] = 3.0 × 10^{-5} M).

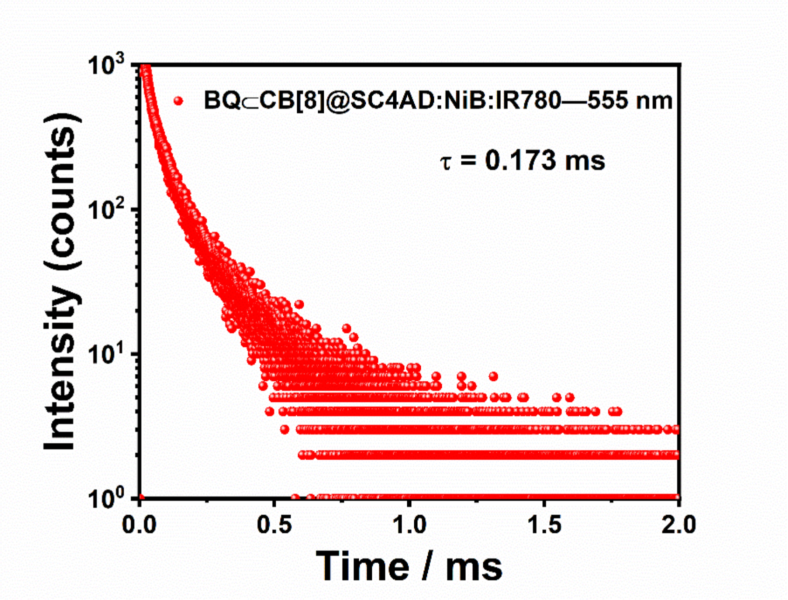

Figure S34. Time-resolved PL decay curves of **BQ**⊂CB[8]@SC4AD:NiB:IR780 at 555 nm in aqueous solution at 298 K ([**BQ**⊂CB[8]] = 3.0×10^{-5} M, [SC4AD] = 1.5×10^{-5} M, [NiB] = 6.67×10^{-7} M, [IR780] = 3.0×10^{-7} M).

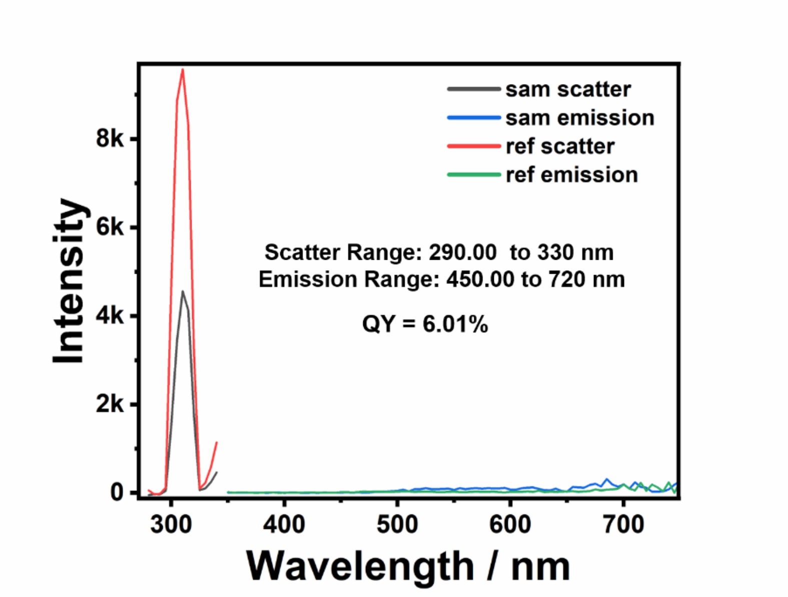

Figure S35. The phosphorescence quantum yield of **BQ**⊂CB[8]@SC4AD:NiB under ambient conditions.

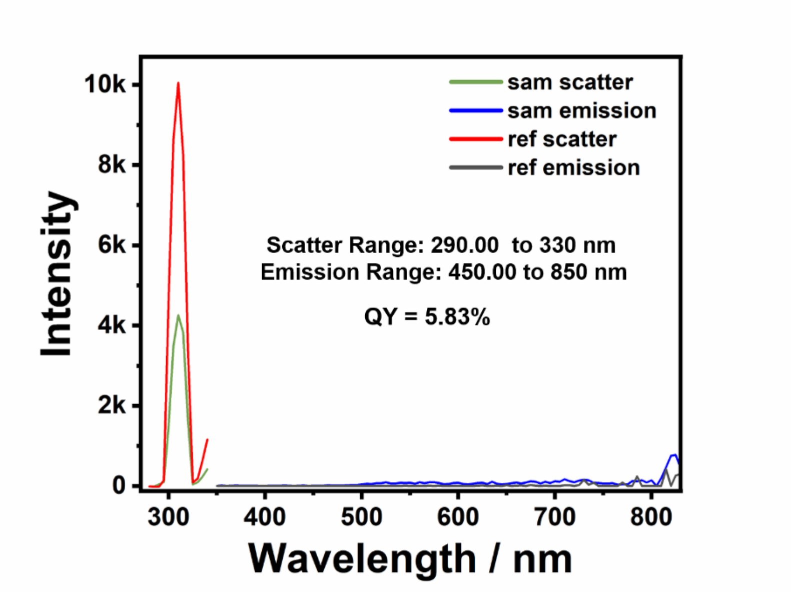

Figure S36. The phosphorescence quantum yields of **BQ**⊂CB[8]@SC4AD:NiB:IR780 under ambient conditions.

The overlap integral, J is a measure for how well donor emission and acceptor absorption overlap and implements the molar absorption $\varepsilon_{\mathrm{A}}(\lambda)$ of acceptor and the area-normalized emission spectra of donor.

The spectral overlap integrals, J^{λ} can be calculated by the following formula[4] :

$$J^{\lambda} = \int \bar{I}_{\lambda}^{\mathrm{D}^*} \varepsilon_{\mathrm{A}}(\lambda)\, \lambda^4 \mathrm{d}\lambda; \int \bar{I}_{\lambda}^{\mathrm{D}^*}\, \mathrm{d}\lambda = 1 \quad \ldots\ldots (1)$$

$\varepsilon_{\mathrm{A}}(\lambda)$ is the molar extinction coefficient of the dopant material; and λ is the energy expressed in wavelength. $I_{\lambda}^{D^*}$ represent the normalized spectral intensity of the donor.

The overlap integrals were calculated to be J_{Cy5} = 6.78 × 10^{15} (dm^3 nm^4)/(mol cm), J_{NiB} = 1.59 × 10^{15} (dm^3 nm^4)/(mol cm).

Energy-transfer efficiency, Φ_{ET}, the fraction of the absorbed energy that is transferred to the acceptor is experimentally measured as a ratio of the fluorescence intensities of the donor in the absence and

presence of the acceptor (I_D and I_{DA}) or a ratio of the lifetime of the donor in the absence and presence of the acceptor (τ_D and τ_{DA}).[5,6]

$$\Phi_{ET} = 1 - \frac{I_{DA}}{I_D} = 1 - \frac{\tau_{DA}}{\tau_D} \qquad \textbf{S1}$$

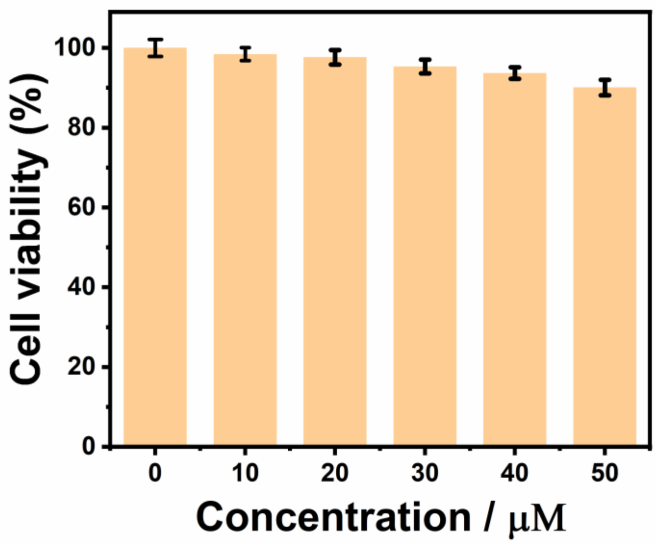

Figure S37. Cell viability of A549 cancer cells for **BQ**⊂CB[8]@SC4AD:Cy5:IR780 at different concentration.

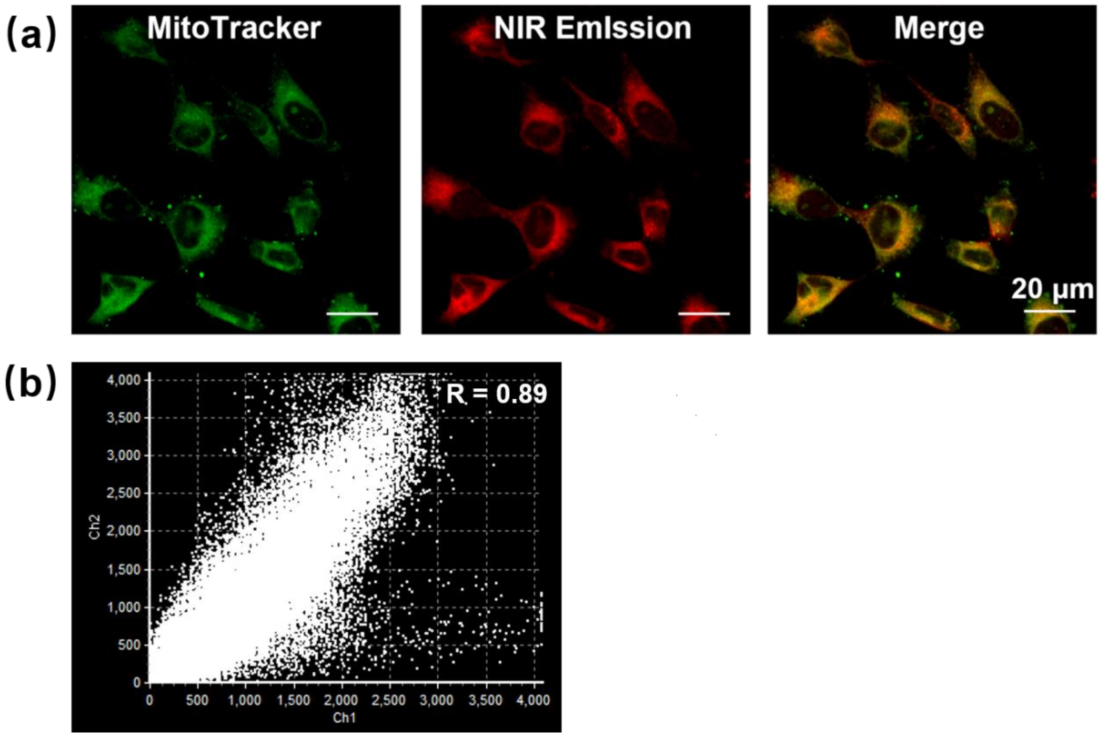

Figure S38. a) Confocal microscopy images of A549 cells co-stained with **BQ**⊂CB[8]@SC4AD:Cy5:IR780 and MitoTracker Green for visualizing the organelle localization. For MitoTracker Green, λ_{ex} = 488 nm, λ_{em} = 500-550 nm. [**BQ**⊂CB[8]] = 3.0 × 10^{-5} M, [SC4AD] = 3.0 × 10^{-5} M, [Cy5] = 1.0 × 10^{-6} M, [IR780] = 3.33 × 10^{-7} M). b) Pearson correlation coefficient obtained from the scatter plot.

Section F. Reference:

[1]. D.-A. Xu, Q.-Y. Zhou, X. Dai, X.-K. Ma, Y.-M. Zhang, X. Xu, Y. Liu, *Chin. Chem. Lett.* **2022**, *33*, 851–854.

[2]. K.-P. Wang, Y. Chen, Y. Liu, *Chem. Commun.* **2015,** *51*, 1647-1649.

[3] G. Yang, J. Tian, C. Chen, D. Jiang, Y. Xue, C. Wang, Y. Gao, W. Zhang, *Chem. Sci.*, **2019**, *10*, 5766–5772.

[4]. A. Kirch, M. Gmelch, S. Reineke, *J. Phys. Chem. Lett.* **2019**, *10*, 310-315.

[5]. X.-M. Chen, Q. Gao, H.K. Bosoyi, M. Wang, H. Yang, Q. Li, *Angew. Chem. Int. Ed.* **2020**, *59*, 10493.

[6]. J.-J. Li, Y. Chen, J. Yu, N. Cheng, Y. Liu, *Adv. Mater.* **2017**, *29*, 1701905.
